# The Dream Catcher experiment: blinded analyses failed to detect markers of dreaming consciousness in EEG spectral power

**DOI:** 10.1093/nc/niaa006

**Published:** 2020-07-15

**Authors:** William Wong, Valdas Noreika, Levente Móró, Antti Revonsuo, Jennifer Windt, Katja Valli, Naotsugu Tsuchiya

**Affiliations:** n1 School of Psychological Sciences and Turner Institute for Brain and Mental Health, Monash University, Melbourne, VIC, Australia; n2 Department of Psychology, University of Cambridge, Cambridge, UK; n3 Department of Psychology, and Turku Brain and Mind Center, University of Turku, Turku, Finland; n4 Department of Cognitive Neuroscience and Philosophy, School of Bioscience, University of Skövde, Sweden, Skövde; n5 Philosophy Department, Monash University, Clayton, VIC, Australia; n6 Department of Perioperative Services, Intensive Care and Pain Medicine, Turku University Hospital, Turku, Finland; n7 Department of Dynamic Brain Imaging, Advanced Telecommunications Research Institute International, Seika, Kyoto Prefecture, Japan; n8 Center for Information and Neural Networks, National Institute of Information and Communications Technology, Suita, Osaka Prefecture, Japan

**Keywords:** NREM sleep, dreams, unconsciousness, EEG correlates, unsupervised machine learning

## Abstract

The Dream Catcher test defines the criteria for a genuine discovery of the neural constituents of phenomenal consciousness. Passing the test implies that some patterns of purely brain-based data directly correspond to the subjective features of phenomenal experience, which would help to bridge the explanatory gap between consciousness and brain. Here, we conducted the Dream Catcher test for the first time in a step-wise and simplified form, capturing its core idea. The Dream Catcher experiment involved a Data Team, which measured participants’ brain activity during sleep and collected dream reports, and a blinded Analysis Team, which was challenged to predict, based solely on brain measurements, whether or not a participant had a dream experience. Using a serial-awakening paradigm, the Data Team prepared 54 1-min polysomnograms of non-rapid eye movement sleep—27 of dreamful sleep and 27 of dreamless sleep (three of each condition from each of the nine participants)—redacting from them all associated participant and dream information. The Analysis Team attempted to classify each recording as either dreamless or dreamful using an unsupervised machine learning classifier, based on hypothesis-driven, extracted features of electroencephalography (EEG) spectral power and electrode location. The procedure was repeated over five iterations with a gradual removal of blindness. At no level of blindness did the Analysis Team perform significantly better than chance, suggesting that EEG spectral power could not be utilized to detect signatures specific to phenomenal consciousness in these data. This study marks the first step towards realizing the Dream Catcher test in practice.

**Highlights**

- The present study is the first reported attempt of the Dream Catcher test.
- Previously reported EEG markers of NREM dreaming were not identified by our blind analyses.
- The correlates of conscious experience may not be detectable in EEG spectral power.

## Introduction

### Background

If we take consciousness to be a natural, biological phenomenon that depends on the neural activity of the brain, then there must be objective patterns of brain activity that directly constitute consciousness and therefore correspond to the subjective features of experience. Whatever type of neural activity consciousness turns out to be, it must be something that incarnates the spatiotemporal patterns of phenomenal experience.

The Dream Catcher test was introduced as an empirical criterion for what would constitute a genuine scientific discovery of the underlying neural constituents of phenomenal consciousness (Revonsuo 2006). The present empirical study, which we call the Dream Catcher *experiment*, is the first attempt to execute a simplified version of such a test. The test itself was originally conceived as an idealized thought experiment, devised to address the explanatory gap that exists between any physical explanation of consciousness and the phenomenal experience of consciousness itself—the so-called ‘hard problem’ (Levine 1983; Chalmers 1995). Arguably, even if one were able to identify the neural correlates of consciousness, those would not suffice to bridge the explanatory gap: *correlations* do not in themselves provide an *explanation* for a phenomenon. Revonsuo (2006) proposed that consciousness would be genuinely explained by the discovery of *constitutive* mechanisms of consciousness at the phenomenal level. [For the crucial distinction between correlates and constituents, see also Revonsuo (2001) and Miller (2015).]

To verify the constitutive mechanisms of consciousness, the Dream Catcher test requires researchers to be able to predict the qualitative features of participants’ phenomenal experience based only on observations of their brain activity, and without access to any information about the participants’ stimulus environment, subjective experience or correlated brain patterns to known perceptual stimuli. This would be achieved by the following proposed stipulations. First, the study of consciousness would be restricted to the domain of sleep, to ensure that the contents of consciousness are largely unrelated to external stimuli. Second, the researchers charged with testing their brain-based model of consciousness would be blinded to participants’ subjective reports, which would be instead recorded by an independent team. These restrictions make the Dream Catcher test a kind of no-report paradigm: a study design aiming to prevent the conflation between processes underlying conscious experience and those underlying the act of reporting conscious experience (Tsuchiya *et al.* 2015). During sleep, the disconnection between external stimuli and subjective experience also prevents the conflation between conscious experience and external stimulus processing, which—while central to waking perception—plays a minimal role in dreams.

Conscious mentation is not just frequent in REM sleep but also occurs throughout the majority of non-rapid eye movement (NREM) sleep (Nielsen 2000; Noreika *et al.* 2009; Nir and Tononi 2010; Windt *et al.* 2016). Recently, specific spectral changes in sleep electroencephalography (EEG) have been found in studies contrasting periods of NREM sleep associated with reports of dreaming against periods without dream recall (Esposito *et al.* 2004; Chellappa *et al.* 2011; Siclari *et al.* 2014, 2017; Scarpelli *et al.* 2017; Siclari *et al.* 2018; Zhang and Wamsley 2019; for a review, see Ezquerro-Nassar and Noreika 2019). These studies concurred that reduced low-frequency EEG power correlates with dream recall, although they neither agreed on the source location of this difference, nor on whether high-frequency activity was also correlated with dream reports.

For our study, the Dream Catcher experiment, we aimed to address the implications of the Dream Catcher test in practice. We have highly simplified the original Dream Catcher test due to the current limitations of neuroscientific knowledge and brain activity measuring capability. Instead of requiring researchers to reconstruct the content of phenomenal experience from a comprehensive set of brain activity data, we introduced a more realistic requirement: to identify the presence vs. absence of dreaming (i.e. the presence vs. absence of conscious experience) from polysomnograms, without access to the self-reports on whether the participants had dreamt or not. Some studies have investigated neural correlates of specific dream content (Dresler *et al.* 2012; Horikawa *et al.* 2013; Horikawa and Kamitani 2017; Siclari *et al.* 2017), but reconstruction of the full phenomenal level would be a step for the distant future.

### Measures of consciousness

Early polysomnography (PSG) consisted of the measurement of voltage fluctuations at various sites of the body, traced onto continuous paper feed, to be interpreted and classified by researchers by visual inspection. Based on features such as the frequency of oscillations at the scalp, intensity of muscle tone activity and type of eye movement, researchers found that they could classify distinct stages of sleep. Remarkably, the REM stage of sleep was found to highly correlate with dream reports, albeit not to be necessary nor sufficient (Aserinsky and Kleitman 1953; Foulkes 1962; Jouvet 1967). Researchers have since increased the array of tools for analysing electrophysiological sleep data, including spectral methods, phase coherence measures and the vast variety of methods devoted to time series analysis in general (Cohen 2014; Arsiwalla and Verschure 2018). Many features of brain electrophysiology have been investigated and reported to correlate with different levels of consciousness. Spectral power differences have been commonly found at characteristic frequency bands; notably, loss of consciousness due to deep sleep and deep general anaesthesia has been associated with increased power at low frequencies [i.e. delta waves, <4 Hz (Noachtar *et al*. 1999)] (Thomsen *et al.* 1991; Hobson and Pace-Schott 2002; Evans 2003; Chellappa *et al.* 2011; Murphy *et al.* 2011; Scarpelli *et al.* 2017; Siclari *et al.* 2017, 2018; Scheinin *et al.* 2018; Zhang and Wamsley 2019). Higher levels of conscious arousal have also been suggested to correlate with a lower spectral exponent (Colombo *et al.* 2019), higher signal entropy or complexity (Bruhn *et al.* 2000; Bein 2006; King *et al.* 2013; Liang *et al.* 2013; Ouyang *et al.* 2013; Liang *et al.* 2015;Sarasso *et al.* 2015; Schartner *et al.* 2015; Hudetz *et al.* 2016; D’Andola *et al.* 2017), stronger phase coherence between brain areas (Nieminen *et al.* 2016; Bola *et al.* 2017; Lee *et al.* 2017; Mikulan *et al.* 2017) and more causally integrated brain areas (Barrett *et al.* 2012; Fasoula *et al.* 2013; D’Andola *et al.* 2017).

With an ever-increasing number of methods, we must be wary that almost surely a proportion of reported effects are false positives. Particularly in cognitive neuroscience and psychology, the high prevalence of unreplicable studies has been a serious issue (Fanelli 2009; Kriegeskorte *et al.* 2009; Vul *et al.* 2009; Schooler 2014). In this regard, a distinguishing feature of the Dream Catcher test is its blinded nature; it prevents biasing researchers towards a certain outcome due to knowing the true conditions of their samples.

### Study design

The Dream Catcher experiment involved two teams. The first team was composed of Valdas Noreika, Levente Móró, Antti Revonsuo and Katja Valli, who designed the Dream Catcher protocol and collected data for the overall experiment—we will call this the Data Team. The second team was composed of William Wong, Jennifer Windt and Naotsugu Tsuchiya, who analysed and classified the brain-based data with restricted access to participants’ dream reports—we will call this the Analysis Team. The team made their classification attempts once for each of five steps of the experiment, where additional information was revealed to them after each step. At the beginning of the experiment, the Analysis Team only knew (i) published details of the Dream Catcher’s data collection method using the early night serial-awakening protocol (Noreika *et al.* 2009), (ii) the scientific literature on dreaming and consciousness published at the time (pre-2018), (iii) the Data Team’s instruction sheet (Supplementary MaterialS4), and (iv) some additional background information from occasional email exchanges with the Data Team regarding the Dream Catcher procedure.

For the Dream Catcher experiment, we focused on dreams occurring within NREM sleep Stages 2–3 in balanced proportions. By contrasting the recorded brain activity between reported dreaming and non-dreaming states in NREM sleep rather than in REM sleep, we expected to better isolate the effect of the presence vs. absence of dreams in the data. Unlike in REM sleep, which has a dream recall prevalence of about 80% (Hobson *et al.* 2000; Nielsen 2000), the frequency of dream reports obtained from Stages 2–3 of NREM sleep is roughly equal to that of non-dream reports (Nielsen 2000; Noreika *et al.* 2009). NREM dreams in general tend to be more fragmented, thought-like, and less vivid than REM sleep dreams (Hobson *et al.* 2000; Mutz and Javadi 2017). There is a contention that the non-vivid sleep mentation in NREM sleep should be categorized separately and that only multimodal, narratively complex and often emotional experiences, which are more typical of REM sleep, should be classified as dreaming (Hobson *et al.* 2000). However, as we were interested in the presence vs. absence of even minimal forms of consciousness, we shall refer to all reports of mentation during NREM sleep as dreams in this article (for a more detailed discussion on this theoretical position, see Discussion section).

Please note the unusual structure of our article, which stems from our complex experimental setup. In the General Methods section, we describe the Data Team’s data collection procedures and the Dream Catcher experiment protocol, and give an overview of the Analysis Team’s strategy for blind classification. The particular procedures and results of the Analysis Team at each blinded step are described in the Blind Classification Methods and Results section. This section chronicles irrevocable decisions made by the Analysis Team over multiple steps of the experiment, and consequently, a number of analyses and procedures are different between each step of blind classification. We close with a discussion of the theoretical and methodological implications of the results for the Dream Catcher paradigm and dream research.

## General Methods

Study design, data collection and the blinding procedure were performed exclusively by the Data Team before any contact with the Analysis Team. The study protocol was approved by the Ethical Board of the University of Turku; all participants signed informed consent following the Declaration of Helsinki. Data collection was conducted at the Sleep Laboratory at the Centre for Cognitive Neuroscience at the University of Turku.

### Participants and data collection

Fifteen Finnish-speaking volunteers were recruited to the study from a larger pool of candidates. They were screened to have no issues with psychological and neurological health, take no central-nervous-system-affecting drugs, or have any sleep disorders at the time of the study. Their handedness was tested by means of the Edinburgh Handedness Questionnaire (Oldfield 1971).

Participants spent one adaptation night in the sleep laboratory, in which the researchers aimed to assess the participants’ sleep latency and ability to give clear dream reports, as well as to familiarize them with sleep laboratory environment. Five participants were excluded following adaptation nights due to sleeping difficulties in the laboratory, unclear dream reports upon awakening from NREM sleep, and/or sleep EEG artefacts due to sweating. The remaining 10 participants spent four experimental nights in the laboratory, for which each participant was compensated with 100 euros in total. One of the participants did not recall any dreams upon awakening from NREM sleep, hence this person’s data were not used in the Dream Catcher experiment. The final data set utilized in the study was collected from nine participants (four males), aged 21–34 years (*M *=* *27, SD = 5.4). Of the nine participants, eight were fully right-handed and one was fully left-handed. Dream reports and PSGs were collected during the first 3–4 h of sleep following an early night serial-awakening paradigm (Noreika *et al.* 2009). Refer to Supplementary MaterialS1 for our sleep data collection procedures and methods, Supplementary MaterialS2 for the interview procedure and Supplementary MaterialS3 for transcribed exemplar dream reports.

### Data selection and blinding

All collected dream reports were divided by two blind raters (Master students in psychology) into four categories following Dement (1955): (i) dreamless sleep, (ii) white dream (i.e. the participant strongly felt they had had some experiences right before awakening, but could not recall any specific content), (iii) uncertain and (iv) dreamful (see Supplementary MaterialS1 for details). For the Dream Catcher experiment, only the (i) dreamless and (iv) dreamful categories were considered. The lowest number of reports from either category from a single participant was three. Following this constraint, three dreamful and three dreamless sleep reports were selected from the nine participants, yielding a pool of 54 reports and the corresponding 1-min pre-awakening EEG segments.

The following criteria were applied in the data selection: (i) Both blind raters should have independently agreed on the basic recall category of the report. (ii) All included dreams should be static (i.e. categories 1–4 of Orlinsky’s Modified Scale for Perceptual Complexity of Dreams; Orlinsky 1962; Noreika *et al.* 2009; Supplementary MaterialS1) in order to reduce the variability of reports. The categories encompass experiences that are either composed of several interconnected perceptual experiences or are short but coherent dreams, the elements of which are integrated into a unified scene. Static dreams were chosen for two reasons. First, static dreams provided us with a relatively homogeneous group of dreamful cases in terms of the phenomenological features of the dreams. Second, static dream reports are much more abundant in early night NREM sleep awakenings, with complex dynamic dreams being more rare. (iii) The 60-s pre-awakening EEG should contain only NREM Stage 2 and/or Stage 3 epochs (i.e. three consecutive 20-s epochs of either or both Stages 2 and 3), scored using Rechtschaffen and Kales’ (1968) guidelines. (iv) The proportions of Stages 2 and 3 pre-awakening epochs across recall categories should not be significantly different within- nor between-participants. (Notably, we scored sleep stages in 20-s epochs rather than the more typical 30-s divisions, thus a mixture of NREM Stages 2 and 3 epochs were often present within a single 60-s pre-awakening EEG segment. We matched the proportions of these stages between dreamful and dreamless sleep conditions. The difference in number of any particular NREM Stage epoch between the dream report conditions for each participant was no more than one, and the pooled number of any particular NREM Stage epoch did not differ between the conditions. See Supplementary Table S5 for the frequency count.) (v) Selected EEG recordings should have the least amount of artefacts, assessed by visual inspection, for each participant.

Regarding the homogeneity of Stage 2 and 3 epochs, it should be noted that slow wave activity (SWA) in general gradually increases during the course of each NREM episode and then rapidly decreases shortly before the beginning of a REM episode (Dijk 2009). We therefore attempted to control for the duration of sleep and particular sleep stages before each awakening. In our data, the time interval between the start of sleep to the first awakening was approximately matched between awakenings that yielded dream reports (*M *=* *7264 s, SD = 3994 s) and awakenings that resulted in reports of dreamless sleep (*M *=* *8026 s, SD = 5369 s; *t*(8) = −0.72, *P* = 0.49, Cohen’s *d *=* *0.16). Furthermore, we woke the subjects up as soon as they had remained in the target sleep stage for 3 to 4 min. There was a non-significant trend towards a shorter duration of sleep before dreamful reports (*M *=* *570 s, SD = 240 s) compared to dreamless reports (*M *=* *685 s, SD = 166; *t*(8) = −1.85, *P* = 0.10, Cohen’s *d *=* *0.56). Nonetheless, the proportion of time spent in specific stages of sleep before awakening was also balanced between dreamless and dreamful conditions as part of fulfilling criterion (iv) of data selection. Generally, SWA also decreases as the night progresses (Dijk 2009). To check whether SWA decreased between serial awakenings during the 3- to 4-h data collection period, we have previously examined whether the EEG spectral power differed between epochs of sleep associated with earlier vs. later awakenings (Noreika *et al.* 2009). We found that power measurements did not differ between them, assuring us that SWA did not differ between sleep epochs associated with earlier and later awakenings of early night sleep.

The blind classification phase of the study was to be undertaken by the Analysis Team. The dataset was blinded using a custom Perl script. This 219-line code loaded the metadata (such as the original recording number, the original participant number, the Session number and the ‘dreamful’ or ‘dreamless’ Condition label) from a comma-separated values file describing the parameters of the 54 samples. The recordings were randomly assigned labels with a consecutive numerical range: a general recording label (ID01–ID54), a participant label (S1–S9), a dreamfulness condition label (C1–C2), a participant-grouped condition label (G01–G18; i.e. 9 Participants × 2 Conditions), and a pairing label (P01–P27; i.e. 9 Participants × 3 Sessions) for pairs of dreamful vs. dreamless recordings from the same participant under the same condition. All these labels were logged into a Microsoft Excel table to be used by the Data Team for evaluating the Analysis Team’s blinded analysis results. Finally, the script output a Windows batch file that renamed the original recording files to their randomized ID01–ID54 file names, to be received by the Analysis Team.

## Blind Classification Methods and Results

**Figure 1.**
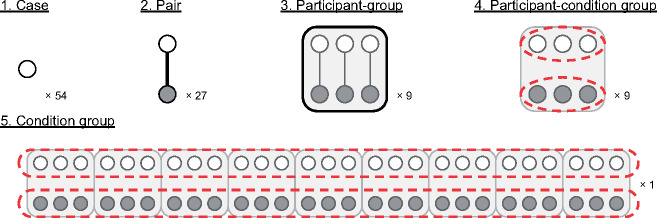
Illustration of blinding information at each step of classification.

We refer to the individual 1-min PSG recordings, given to the Analysis Team, as ‘cases’. In Table 1, we present a review of what information was given to the Analysis Team at each step of this blind classification task, as well as the terms we use in this article to refer to the various groupings of the cases revealed during the experiment. The terms are also illustrated in Fig. 1.

**Table 1.** Blinding of information at each step of classification

Step no.	Collective name for data	Available case information to Analysis Team			
1	‘Case’				
2	‘Pair’	Pairwise: dreamful vs. dreamless, same participant.			
3	‘Participant-group’	Pairwise: dreamful vs. dreamless, same participant.	Groups of six cases: same participant.		
4	‘Participant-condition group’	Pairwise: dreamful vs. dreamless, same participant.	Groups of six cases: same participant.	Groups of three cases: same dreamfulness, same participant.	
5	‘Condition group’	Pairwise: dreamful vs. dreamless, same participant.	Groups of six cases: same participant.	Groups of three cases: same dreamfulness, same participant.	All dreamful vs. all dreamless.

### Overall strategy

The methods described in this section were devised independently of the Data Team. The Analysis Team approached the classification problem firstly as a clustering problem. They assumed that brain states would be more homogeneous during non-dreaming than during dreaming, because various contents of dreaming would possibly diversify brain states. Such homogeneity would be amenable to cluster analysis, which is ideally suited to discover and group observations of high similarity in an objective manner, based on extracted features of the data. The Analysis Team would only need to make a subjective determination of dreamfulness in line with previous findings once two clusters were produced (a choice with only two alternatives).

The Analysis Team operated primarily in the Matlab software environment (The MathWorks, Inc. 2012); some of the EEG data handling was facilitated by the EEGLAB toolbox in Matlab (Delorme and Makeig 2004). We will specify otherwise where relevant.

### Clustering method

**Figure 2.**
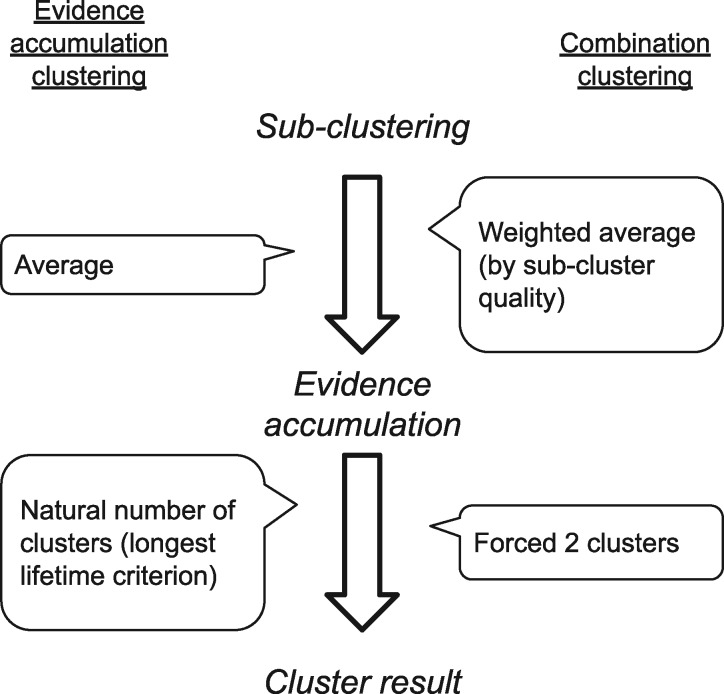
Contrast between the methods, evidence accumulation clustering (Fred and Jain 2005) and combination clustering (Wong and Tsuchiya 2020).

For clustering algorithms, the Analysis Team chose an evidence accumulation clustering (EAC) approach (Fred and Jain 2005) with modifications. It was chosen over more common clustering techniques, such as *k*-means clustering and hierarchical clustering, for its demonstrated improved ability to identify clusters of arbitrary shapes and sizes. Throughout the blind classification procedure, the Analysis Team clustered cases into two groups based on the similarity of extracted features. For the specific purposes of this study, they made changes to EAC. The modified method was called ‘combination clustering’. See Fig. 2 for a comparison; details in Wong and Tsuchiya (2020); see also Supplementary MaterialS6. Here, we provide only a cursory description of combination clustering.

EAC works by accumulating the results of multiple clusterings—evidence—of the same data. Evidence consists of multiple runs of simpler clustering algorithms, where each may give incomplete but diversified information about similarities in the data. Here, we shall call the producing of pieces of evidence *sub-clusterings* so as not to confuse them with the hierarchical clustering procedure that follows the *evidence accumulation* step. In Fred and Jain’s (2005) experiments, the authors implemented sub-clustering using multiple, randomly seeded runs of *k*-means clustering (MacQueen 1967) and then counted the frequency of co-association between each pair of data points. In the evidence accumulation step, these sub-clustering results were tallied to produce the co-association similarity matrix, upon which they performed evidence accumulation via hierarchical clustering, with either single- or average-linkage criterion (Sokal and Michener 1958). They finally thresholded the linkage distance so as to obtain the natural number of clusters present in the data.

The Analysis Team modified EAC for three reasons. First, unlike in the original EAC, the team knew from the outset that they were dealing with two equal-sized clusters. They therefore modified the final step from finding the natural number of clusters in the data to finding just two clusters. However, especially for noisy data sets, a linkage threshold that produces two clusters often produces ones of asymmetric sizes, wherein the smaller cluster may consist of one or a few outliers. To cope with this problem, the Analysis team chose the highest threshold that produced at least one cluster that is as close to 50% of the cases as possible, and all other clusters were reclassified as belonging to the other class. This forced the clustering results to produce two classes of close-to-equal sizes, where at least one of them was guaranteed to contain cases with similar (i.e. more homogeneous) features; the Analysis team postulated that those may represent dreamless cases, however they classified the clusters based on their features rather than their homogeneity.

Second, as the feature spaces consisted of up to 2475 dimensions (or features), this far exceeded the maximum of 64 dimensions demonstrated by Fred and Jain (2005). To avert a possible curse-of-dimensionality problem (see Bellman 1957), the Analysis team limited the number of considered features for each run to no more than nine at a time. They found that this modification made the method more robust when clustering noisy features in experiments with synthetic data (Wong and Tsuchiya 2020). The clustering ensemble in combination clustering was therefore populated with sub-clusterings based on many different *combinations* of features.

Third, to give greater weight to more meaningful sub-clusterings during evidence accumulation, the Analysis Team also introduced weighting of sub-clustering results by a goodness-of-clustering metric: the mean silhouette value. [See the method paper by Wong and Tsuchiya (2020) for omitted details about the modifications introduced in combination clustering.]

The combination clustering algorithm consisted of further modifiable subcomponents, including sub-clustering, weighting and hierarchical clustering. We will describe the specific methods used in each step as they appear.

### Step 1

#### Method

The task in the first step of blind classification was to classify the 54 blinded 1-min polysomnograms—referred to as *cases*—for dreamfulness. Data included simultaneous 25-channel EEG, 2-channel electrooculography (EOG) and 2-channel electromyography (EMG); the channels’ nominal locations were provided. At this step, minimal information was known about the data that could be used for classification. The Analysis Team began with an initial exploration of feature extractions, followed by clustering based on a focussed set of features using the combination clustering method (Wong and Tsuchiya 2020).

First, the Analysis Team considered eight different methods of analysis to obtain features to be used for combination clustering, based on the previous EEG literature on loss of consciousness (e.g. sleep, anaesthesia and brain injury), listed with detailed methods in Supplementary MaterialS7. These were spectral power at established frequency bands, spectral power at fine frequency resolution, autocorrelation features as described by Thomsen *et al.* (1991), permutation entropy (Bandt and Pompe 2002; Ouyang 2012), approximate entropy (Pincus 1991; Pincus *et al.* 1991; Lee 2012), EOG root mean square (RMS) activity, EMG RMS activity and spectral power in temporo-occipito-parietal areas as described by Siclari *et al.* (2014). (Siclari *et al.*’s (2014) preprint article contained differences from Siclari *et al.*’s (2017) article. The Analysis Team utilized the findings reported in the preprint until Step 5, when the print article was finally published in a peer-reviewed journal.) As utilizing too many features would be expected to result in overfitting (Domingos 2012), the Analysis Team aimed to select only a few features best suited for the proper clustering procedure.

In the absence of any ground truth, the Analysis Team sought features that would produce clustering behaviour expected of correct classification of dream report conditions. They operationalized this by how consistently certain features clustered cases when the cases were split into four 15-s time segments. (The concept of consistency as used in this context should only be taken to mean the consistency of the clustering results across time.) Promising features would produce (i) clustering results that were consistent across temporally adjacent segments of time or (ii) results that were increasing in consistency for time segments more proximal to the time of awakening. A consistency metric between the clusterings of any two segments was formulated as follows.

Firstly, let us denote each case as *D*(*i*) (where *i *=* *1, 2, …, 54), and divide it into four segments as *D_j_*(*i*) (where *j *=* *1, 2, 3, 4). Clustering would assign to each *D_j_*(*i*) a membership label for one of two clusters: *c*1 and *c*2. Importantly, these clusters were not classified for dreamfulness; thus, *c*1 in one segment can correspond to either *c*1 or *c*2 in any other segment. But, because we always labelled the data by one of two clusters, there are only two possible ways to map *c*1 from one segment to *c*1 from another. Let us denote the proportion of cases that remain in the same cluster between segment *m* and *n* under the first and second mappings as *π*_1_(*m*, *n*) and *π*_2_(*m*, *n*). Then, the equality *π*_1_(*m*, *n*) + *π*_2_(*m*, *n*) = 1 holds for any clustering result. We thus define consistency of clustering between segment *m* and *n* as follows:

(1) Cm,n=2×π1m,n-0.5=2×π2m,n-0.5,

in which *C*(*m*, *n*) takes the value 0 when the clustering is not consistent at all, and 1 for perfect consistency.

The Analysis Team performed clustering on all cases for each time segment separately, and with each of the eight methods of feature extraction. Based on their resulting consistency measures, they chose to classify dreamfulness based on the *PowerFine* feature set (detailed in Supplementary MaterialS7) for Step 1.

Briefly, the *PowerFine* feature set consisted of cases’ power spectral density (PSD) estimates for each EEG electrode, in frequency bins between 0 and 49.5 Hz in 0.5 Hz steps (i.e. 99 features per electrode). The feature set in total had 2475 features for each case (99 × 25 electrodes). Note that PSDs were estimated throughout this study using fast Fourier transform and Welch’s method (Welch 1967) using Hann windows with 80% overlap.

The Analysis Team performed the sub-clustering stage of combination clustering 82 475 times; 2475 sub-clusterings were performed corresponding to each unique feature, and 10 000 random combinations of features were sub-clustered for each number of features *k* between 2 and 9 inclusively. Based on the result of the above procedure, the Analysis Team contrasted each feature between the two clusters by taking the Cohen’s *d* effect size (Cohen 1988) of their values after log-transformation. Cohen’s *d* is calculated as

(2) d=μ1-μ2σ,

where the term *μ_1_* − *μ_2_* is the difference between the clusters’ means, and *σ* is their pooled standard deviation. The Analysis Team finally classified the cluster with overall higher low-frequency activity and lower high-frequency activity to be from the dreamless condition, concordant with Siclari *et al.*'s (2014) findings.

#### Results

**Figure 3.**
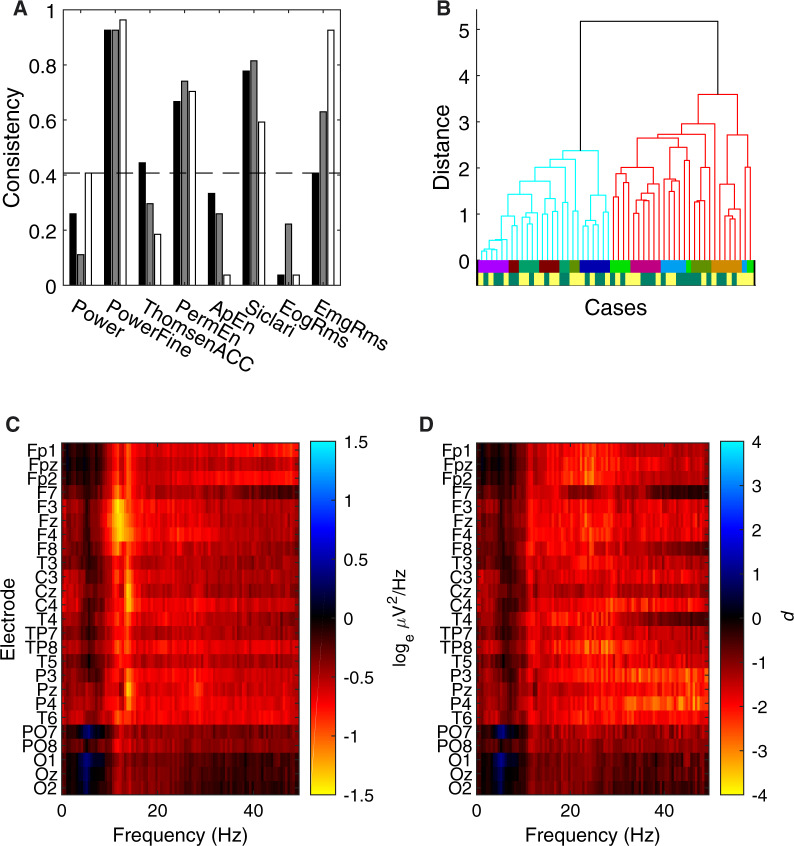
Step 1 blind classification results. (**A**) Temporal consistency for each of the eight candidate feature sets. The consistency measure quantified the degree of agreement of unsupervised clustering between two consecutive 15-s segments of data; a consistency of 1 corresponded to identical clustering results. The black, grey and white bars respectively correspond to the consistencies between segment 1 and 2, 2 and 3, and 3 and 4. The dashed line is the upper bound of the 95% CI for the null model, computed by Monte Carlo simulation, with Bonferroni correction. The Analysis Team decided to use the *PowerFine* feature set for Step 1 classification based on this result. (**B**) Dendrogram of clustering in the *PowerFine* feature set. The two clustered branches are differentially coloured bright cyan (Cluster 1) and darker red (Cluster 2). Cases were hierarchically clustered using average-linkage, following evidence accumulation of their pairwise coassociation similarity. The bottom two colour-coded rows are the true Participant identities (only revealed to the Analysis Team after Step 2) and Dreamfulness identities (only revealed after Step 5), respectively; cases with the Dreamful condition are in dark green, and Dreamless in bright yellow. (**C**) Mean difference in power spectra (Cluster 2 subtracted by Cluster 1) for all 25 EEG channels as a heat map. The colour scale is in units corresponding to the natural logarithm of μV2/Hz. (**D**) The effect sizes of the difference in *C*, quantified in Cohen’s *d*.

The temporal consistency results are summarized in Fig. 3A. The *PowerFine* candidate feature set exhibited highest consistency (>0.9) for three pairs of consecutive time segments (i.e. 1 vs. 2, 2 vs. 3 and 3 vs. 4). This meant that class memberships of two cases tended to be consistent across four 15-s time segments, despite clustering being performed completely independently across segments. This and three other candidates were found to be significantly consistent following Bonferroni correction for multiple comparisons (two-tailed Binomial test, *N *=* *54, unadjusted *P* ≤ 0.001), including feature sets *PermEn*, *Siclari* and *EmgRms*. With regard to positive trends in temporal consistency within individual participants, only *EmgRms* exhibited a difference between the temporal consistencies of the first half and last half (one-tailed permutation test, Bonferroni-corrected *P* = 0.0002).

Using the *PowerFine* feature set, the Analysis Team performed combination clustering and obtained two clusters with unequal membership numbers (28 vs. 26). The dendrogram in Fig. 3B gives a visualization of the separation between the clusters. Figure 3C shows the mean difference in power by Cluster 1 subtracted from Cluster 2, and Fig. 3D shows its effect size in Cohen’s *d*. The Analysis Team observed generally lower high-frequency power (ca. 11–30 Hz) in the average of Cluster 1 compared to Cluster 2 (visible in heat maps Fig. 3C–D). Thus, based on Siclari *et al.* (2014)’s findings, the Analysis Team interpreted Cluster 1 to contain more dreamless report cases, and Cluster 2 to contain more dreamful ones. (To see the exact case-wise classification for this and all proceeding steps, please refer to Fig. 9.) The Analysis Team submitted this classification, and the Data Team determined the accuracy to be 54%, which was not significantly different from chance according to a two-tailed Binomial test (*N *=* *54, *P* = 0.68).

The Analysis Team’s clustering turned out to strongly group for participant identity and not for dreamfulness. Although full participant information was not revealed until Step 3, we show them in Fig. 3B below the dendrogram by the upper row of coloured lines, whose colours code for each of the nine participants. The fact that clustering strongly grouped for participant identity was nonetheless determinable by the Analysis Team in Step 2: with the revelation of pairing information, it was found that 24 of the 27 pairs of cases were wrongly classified as co-occurring in the same cluster.

### Step 2

#### Method

Together with the announcement of the result for Step 1, the Data Team removed the first layer of blindness by revealing the grouping of the cases into 27 *pairs*. Each pair consisted of one dreamful and one dreamless case from the same participant. Still employing the combination clustering method, the Analysis Team was guided by a simple formalism of the linear mixed model

Cases ∼ Dreamfulness+(1 | Participant),

where the observed data (*Cases*) should reflect the main effect (*Dreamfulness*) with added random effects (1 | *Participant*). Assuming that the main effect of dreamfulness would be constant across participants, the Analysis Team treated the 54 cases as 27 single observations, each with feature values taken as the difference between a given pair. If these assumptions were correct, one can see that successful classification would be obtained through minimization of the variance across paired cases (i.e. alignment of feature vectors along the average difference between the two classes).

Also at this step, the Analysis Team reconsidered the features to use for clustering. In the previous step, their feature set consisted of 2475 features of PSDs across the scalp, which they suspected in hindsight to have been excessively numerous and thus contributed to poor classification performance due to overfitting data (Domingos 2012). They also suspected that the clustering algorithm might have performed better with a more encompassing feature set than one that only looked at EEG. To address these issues, the Analysis Team both condensed the EEG PSD features into a smaller number and expanded the diversity of features that composed the feature set for classification. (See Supplementary MaterialS8 for details.)

In brief, because the spectra across electrodes were apparently similar (see Fig. 3), the Analysis Team averaged the PSDs across all electrodes in 19 frequency bins that were logarithmically spaced—thus reducing the number of features from 2475 to 19. As for the lost locality information, they delegated this to a focused set of 11 features based on the hot zone findings reported by Siclari *et al.* (2014), which were accessible as a preprint manuscript at that point in time. These features included the whole-brain power at low- and high-frequency bands, low-frequency parieto-occipital power at various time windows, high-frequency frontal power at various time windows, and high-frequency power at hot zones relating to various perceptual categories. Lastly, the Analysis Team included 20 features extracted from the time course of EMG and EOG, computed differently from Step 1. For each 30-s segment of EMG or EOG, they computed the RMS values of all consecutive 1-s time windows, and took the 0th, 25th, 50th, 75th and 100th percentiles of those as features. This resulted in 10 features (5 percentiles × 2 segments) for each modality. In total, this set consisted of 50 features (19 scalp-average PSD + 11 *Siclari* features + 10 EMG + 10 EOG).

The difference between each pair was extracted by their subtraction in each feature value after Studentization. Because the Analysis Team did not know which case of the pair belonged to which dream report condition, the polarity of the difference was arbitrarily assigned. As a result, they obtained 27 real-valued vectors in 50 dimensional feature space, whose *orientation*—and, importantly, not direction—represented the difference between the pairs’ features.

**Figure 4.**
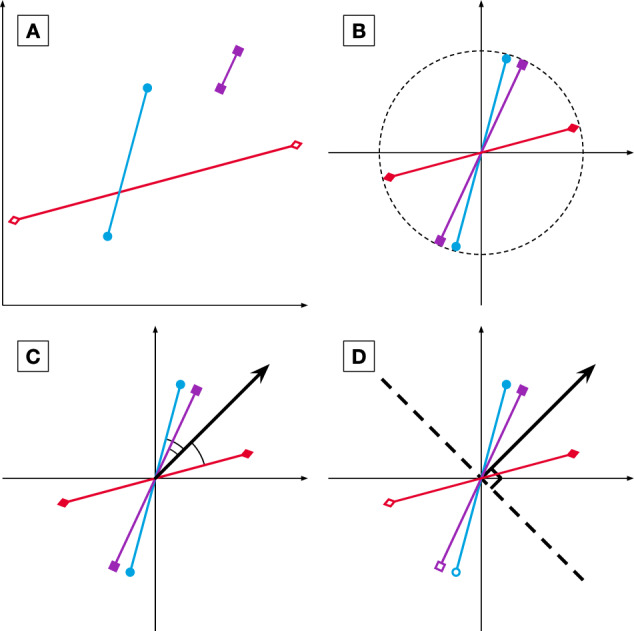
Step 2 pairwise sub-clustering schematic. Here, cases are depicted as points in a 2D feature space; pairs are represented as two cases joined by a line. The basic concepts of pairwise sub-clustering begin in (**A**), with the pairs of cases producing each a vector quantifying the difference between a dreamful case and a dreamless case. These difference vectors are then normalized in (**B**) to have equal length and intersect with the origin at their midpoints. Next, the mean orientation among all difference vectors is estimated in (**C**), defined as a vector whose orientation maximizes the average absolute cosine similarity between it and the difference vectors. The mean orientation vector estimates the true qualitative difference between dreamfulness and dreamlessness. It is depicted in the figure as a bold arrow originating from the centre point. Finally, the cases are sub-clustered in (**D**) by introducing a hyperplane normal to this vector (bold, dashed line) and labelling the cases falling on each side of the hyperplane as co-associating. Paired cases will never co-associate.

For the combination clustering procedure of this step, sub-clustering was performed on the above-transformed representation of the pairs, as follows. First, the feature space was subsampled to the combination of features to be used by the sub-clustering. Figure 4A shows a mock example where two features were chosen, thus representing the difference vectors (of which there are three, for illustrative purposes) in two dimensions. Next, each vector was centred at their midpoints and normalized to have unity length (Fig. 4B). A new vector representing the average orientation among all the difference vectors was then found (Fig. 4C), called the mean orientation vector, which should maximize the mean absolute cosine similarity between each difference vector and itself. Its orientation estimates the true qualitative difference between dreamfulness and dreamlessness. A hyperplane was then drawn normal to this vector, intersecting the origin (Fig. 4D). This hyperplane splits each difference vector in two, which finally allowed those corresponding cases falling on each side of the hyperplane to be sub-clustered together.

For the goodness-of-clustering values—used to weight each sub-clustering within combination clustering—the Analysis Team took the mean absolute cosine similarity. In order to make these values comparable between sub-clusterings of different dimensionalities, the mean absolute cosine similarity was also divided by the expected value of such if the difference vectors were randomly oriented. This was computed using Monte Carlo simulations, where the number of difference vectors and the dimensionality were matched to those of each sub-clustering.

#### Results

**Figure 5.**
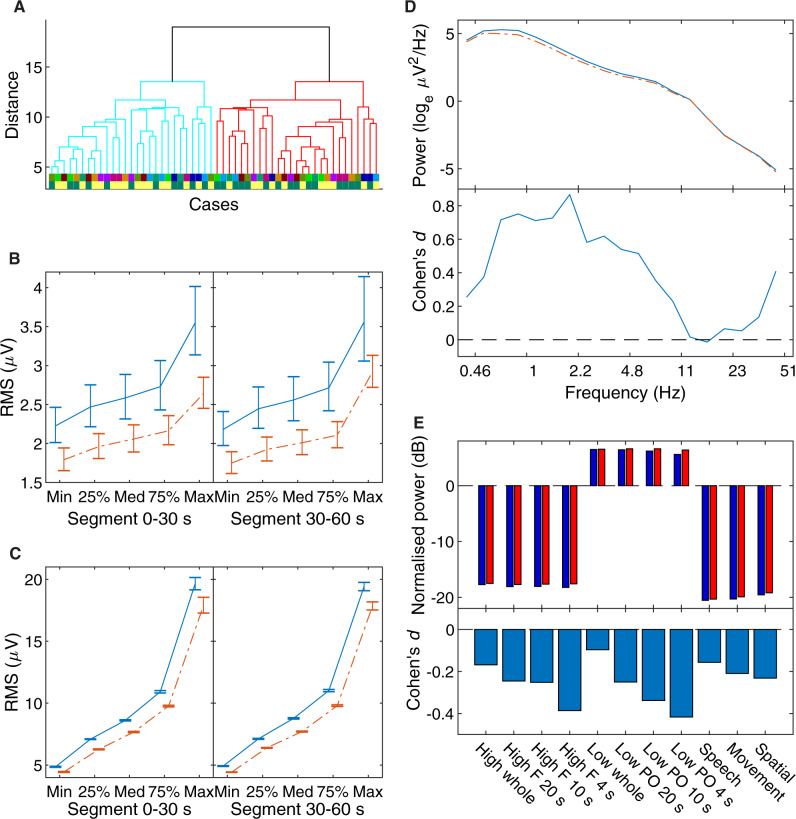
Step 2 blind classification results. (**A**) Dendrogram of clustering, in the same format as in Fig. 3A. Cases were hierarchically clustered using average-linkage, following evidence accumulation of their pairwise co-association similarity. (**B**) The EMG feature means and standard errors of Cluster 1 (solid blue line) and Cluster 2 (broken red line); as these features were extracted from one of two time segments, their means are shown separately in two panels. These statistics were calculated from log-transformed data. (**C**) The same for EOG activity. (**D**) The EEG power spectral density average of Cluster 1 and Cluster 2, and their effect sizes. For the top panel, Cluster 1 is the solid blue line, and Cluster 2 is the broken red line. In the bottom panel, their statistical differences are expressed as Cohen’s *d*, in lieu of error bars for the top panel. (**E**) The difference in power for frontal (‘F’) and parieto-occipital (‘PO’) electrodes, separately for ‘high’ (18–50 Hz) and ‘low’ (1–12 Hz) frequencies, over the indicated period of time just before awakening; and for the whole scalp locality (‘whole’) over 20 s just before awakening. For the top panel, Cluster 1 in dark blue bars, and Cluster 2 in bright red. Like for D, their statistical differences are expressed in the bottom panel as Cohen’s *d*.

The Analysis Team obtained two equally sized clusters following the clustering procedure. The dendrogram in Fig. 5A gives a visualization of the separation between the clusters. Differences were found in the mean of their features most prominently in EOG activity (Fig. 5*C*); the cluster with higher EOG activity (Cluster 1) also had higher EMG activity (Fig. 5*B*) and low-frequency EEG activity (Fig. 5*D*). Differences in *Siclari* hot zone features were small and otherwise inconsistent (Fig. 5*E*). Although major differences were manifest in the EOG and EMG features, the Analysis Team had no rationale based on the literature for using them to determine which cluster corresponded to dreamfulness. Thus, based on EEG differences, they interpreted Cluster 1 to contain more dreamless report cases, and Cluster 2 to contain more dreamful report cases. The Data Team determined the accuracy of this classification to be 59%, which was not significantly different from chance (two-tailed Binomial test, *N *=* *27, *P* = 0.44). Unlike after Step 1, the Analysis Team did not gain much new information from the feedback on their performance and the newly revealed participant identities.

### Step 3

#### Method

The Data Team removed the second level of blindness with the revelation of cases grouped by common participants. This resulted in nine distinct *participant-groups*, each associated with six cases, which were composed of three condition-balanced pairs of cases. Given this information, the Analysis Team exploited participant information by removing participant-specific, condition-irrelevant components from the EOG-EEG signals. To this end, they utilized independent component analysis (ICA), a technique for ‘unmixing’ multichannel time series into their underlying, statistically independent time series components (Gävert *et al.* 2005). They afterwards recomposed the case recordings after selecting and removing the condition-irrelevant components, which might have consisted of artefacts caused by eye and muscle movements. The full details of the methodology are described in Supplementary Material S8.

From the recomposed cases of all participants, the Analysis Team extracted the same set of features explained in Step 2 and performed combination clustering on it. A different set of combination clustering sub-clustering procedures was devised to take into account participant information. Taking the method of Step 2’s pair difference vector sub-clustering by mean orientation, the team sub-clustered the cases in four ways. In two of the ways, similar to Step 2, they sub-clustered cases in a pairwise manner: firstly with respect to the mean orientation amongst all pairs, and secondly with respect to the mean orientation of their own participant. The other two ways used the method of Step 1’s *k*-means sub-clustering and sub-clustered the unpaired cases: firstly amongst all cases, and secondly amongst each participant-group of cases. Therefore, there were four ways in which they performed sub-clustering. Supplementary Fig. S8.2 gives an overview of the scheme. They calculated the final similarity matrix as the average of these four different sub-clustering methods’ results; the exact details are also described in Supplementary MaterialS8. The resulting clusters produced with this method were thereafter classified for dreamfulness using the same rationale as in Step 1.

#### Results

**Figure 6.**
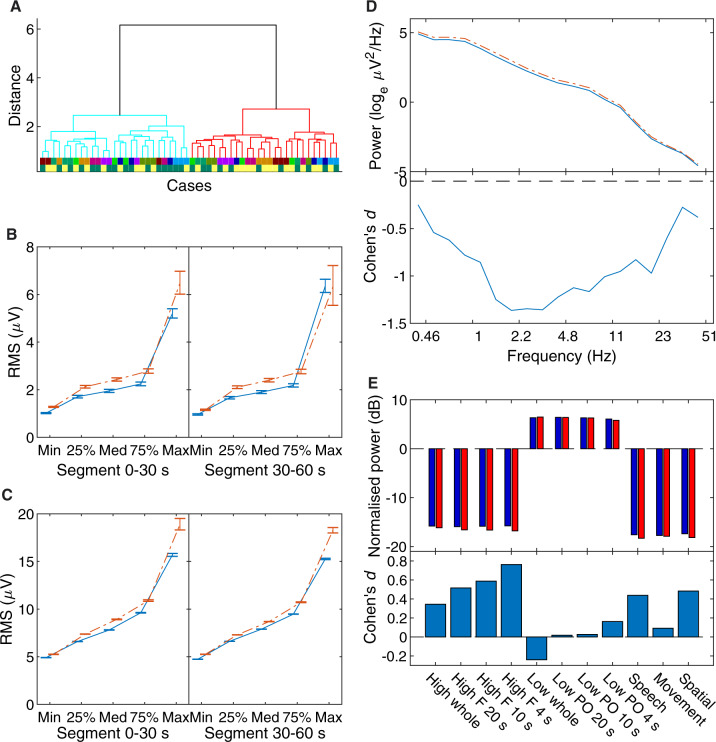
Step 3 blind classification results. The same format as in Fig. 5. Cases were hierarchically clustered using Ward’s minimum variance linkage after evidence accumulation.

The Analysis Team obtained two equally sized clusters following the clustering procedure, using Ward’s method (Ward 1963) as an alternate hierarchical linkage measurement because the average-linkage method, used in the previous steps, resulted in clusters of uneven sizes, which was undesirable. Figure 6 shows results for this step in the same format as Fig. 5 for Step 2. The Analysis Team found differences between the clusters most prominently in EOG activity (Fig. 6C); the cluster with higher EOG activity (Cluster 2) also had higher EMG activity (Fig. 6B) and low-frequency EEG activity (Fig. 6D). In contrast, differences in *Siclari* hot zone features were small (absolute effect size Cohen’s *d *<* *0.7) and otherwise inconsistent with each other in regard to their reported interpretations (Fig. 6E). Faced with similar results to Step 2, the Analysis Team interpreted Cluster 1 to contain more dreamful report cases (see Fig. 9 for exact case-wise classification).

The Data Team determined the accuracy of this classification to be 59%, which was not significantly different from chance (two-tailed Binomial test, *N *=* *27, *P* = 0.44). This feedback also did not give the Analysis team any further insights into their classification.

### Step 4

#### Method

The third level of blindness was removed by grouping all cases with the same dream report condition from each participant. This effectively gave each participant two unlabelled condition groups of three cases each—referred to as *participant-condition groups*. The Analysis Team approached classification similarly to Step 3, first removing condition-irrelevant components using ICA with an adjusted component removal procedure, but then taking the difference in features between the average of conditions for each participant as observation vectors. This resulted in nine 50D vectors on which sub-clustering was performed in a pairwise manner. See Supplementary MaterialS8 for full details. Following combination clustering, the team classified the clustering result for dream report conditions according to the rationale set out in Step 1.

#### Results

**Figure 7.**
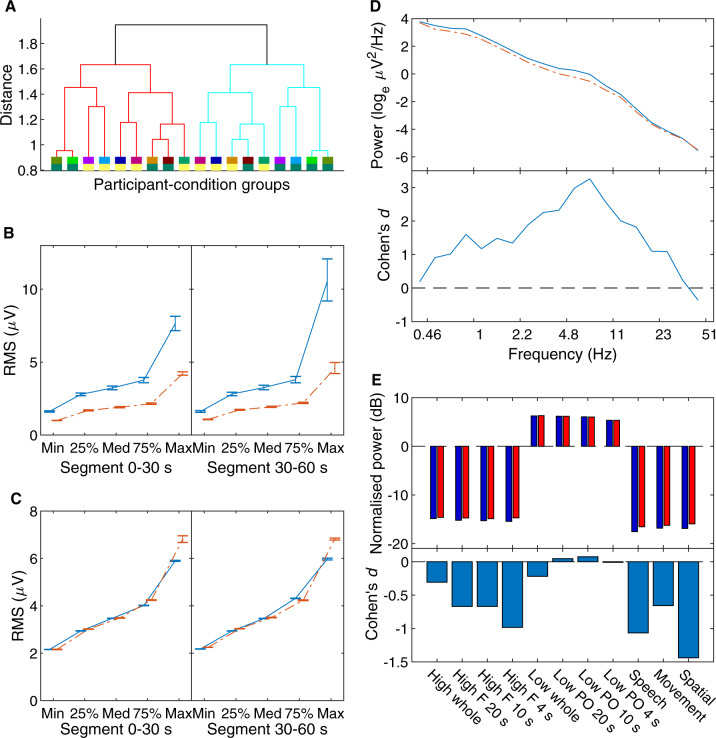
Step 4 blind classification results. The same format as in Fig. 5 besides (**A**), which shows the dendrogram on participant-condition group averages.

Figure 7 reports the results in the same format as in Figs 5 and 6. However, Fig. 7A shows the dendrogram of clustering at only the participant level, unlike in Figs 5A and 6A, as they were already grouped at this Step. Unlike Steps 2 and 3, the Analysis Team found a prominent difference between the clusters in EMG activity (Fig. 7B) but not EOG activity (Fig. 7C); the cluster with higher EMG activity (Cluster 1) also had higher low-frequency EEG activity (Fig. 7D). Differences in *Siclari* hot zone features also indicated lower frontal high-frequency activity for this cluster (Fig. 7E), which in their study indicated an absence of dreaming experience. The team thus interpreted Cluster 1 to contain more dreamless report cases and Cluster 2 to contain more dreamful report cases (see Fig. 9 for exact case-wise classification). The Data Team determined the accuracy of this classification to be 44%, which was not significantly different from chance performance (two-tailed Binomial test, *N *=* *9, *P *=* *1).

### Step 5

#### Method

The penultimate layer of blindness to be removed was information on the common dream report condition across all cases. This resulted in just two groups—one from the dreamful condition, the other from the dreamless condition—each consisting of 27 cases fully labelled by common participants. No clustering was required. To make their final blind classification, the Analysis Team replicated three measures based on the significant differences reported by Siclari *et al.* (2017) and Scarpelli *et al.* (2017), both of which had been published around the time when the Analysis team conducted the Step 5 analysis. (Note that this was the first time the Analysis Team utilized Siclari *et al.*'s 2017 findings. Previous to this, findings based on the 2014 preprint article were used.) Two of the features were Siclari *et al.*’s low and high frequency hot zone power, named respectively *SBP low* and *SBP high* (SBP for the initials of the first three authors of the Siclari *et al.* paper), and one feature was the low-frequency activity reported by Scarpelli *et al.* named *Scarpelli* (see Supplementary MaterialS8 for full details). The Analysis Team classified these condition groups for dream report condition based on the group-average of these features.

#### Results

**Figure 8.**
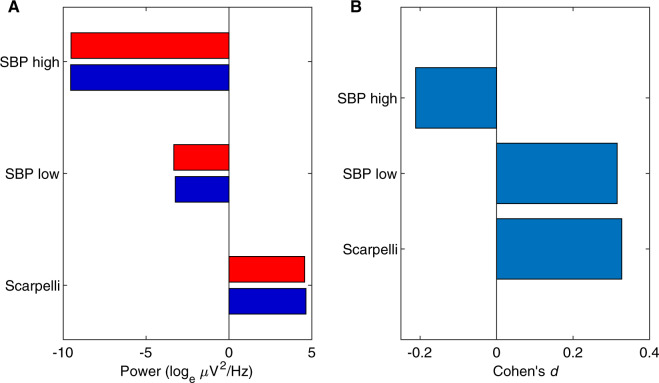
Step 5 blind classification results. (**A**) The difference in feature means between Cluster 1 and Cluster 2. Cluster 1 in dark blue bars and Cluster 2 in bright red. (**B**) The effect sizes of the difference in A, of Cluster 1 from Cluster 2 in Cohen’s *d*, after subtracting each participant’s means.

**Figure 9.**
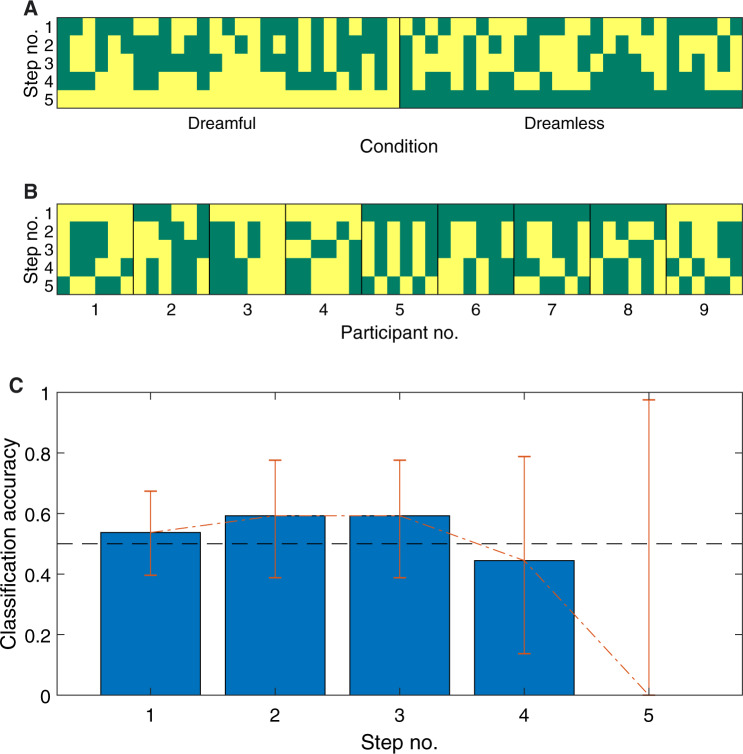
Case classifications through five levels of information. In (**A**) and (**B**), the dark green cells are cases that were classified as *dreamful*. Cases are horizontally sorted by Condition in *A* and Participant Number in B. In (**C**) the error bars are 95% confidence intervals; chance-level accuracy (50%) is demarcated by the dashed, black line. See also Table 2.

The difference in feature means after removing inter-participant variability is shown in Fig. 8A. All features had low effect sizes (absolute Cohen’s *d *<* *0.4). As they were consistent in their indication of dream report condition, the Analysis Team interpreted Cluster 1 to contain more dreamless report cases and Cluster 2 to contain more dreamful report cases (see Fig. 9 for exact case-wise classification). The Data Team determined this classification to be inaccurate.

## Discussion

The Dream Catcher test is a paradigm to examine whether an understanding of the neural constituents of consciousness (i.e. experiences) is indeed genuine, by separating the measurement of brain activity from associated subjective reports. The test should be performed under the stipulation that the full contents of consciousness are generated internally by neural mechanisms operating spontaneously and independently of external stimulation. If a scientist can reliably reconstruct the full contents of consciousness based only on observations of the neural activity and without access to information on external stimuli or subjective reports, then the test is passed.

We executed a simplified version of the Dream Catcher test with data from nine participants, collected by the Data Team with an early night serial-awakening paradigm. The Analysis Team’s task was to sort the EEG segments (i.e. 60-s PSGs preceding awakenings and interviews) into two groups: segments associated with dream reports vs. segments associated with reports of non-dreaming. The Data Team evaluated the Analysis Team’s performance over five iterations with gradual removal of blindness (Fig. 1 and Table 1). They were: (i) all cases unlabelled, (ii) cases paired by complementary dreamfulness conditions from the same participant, (iii) cases labelled by common participant, (iv) cases labelled by common dreamfulness condition and participant and (v) cases labelled by common dreamfulness condition. The Analysis Team approached the classification task by clustering together quantitatively similar cases into two groups and then manually classifying those groups based on findings from the recent literature. The similarity metrics used in clustering were based on features extracted from power and location of EEG spectra, and EMG and EOG activity.

At all levels of blindness, the Analysis Team was unable to correctly classify between dreamfulness vs. dreamlessness with statistically significant accuracy (Fig. 9 and Table 2); the best performance was achieved at Steps 2 and 3 with an accuracy of 59% (*P* = 0.44). Thus, the Analysis Team did not pass even this rudimentary form of the Dream Catcher test.

**Table 2.** Summary of classification performance through five levels of information

Step	Information revealed	Number of decisions	Number correct	Accuracy (%)
1	Case	54	29	54
2	Pair	27	16	59
3	Subject	27	16	59
4	Subject-condition	9	4	44
5	Condition	1	0	0

None of the steps resulted in accuracies that were statistically significant (*ɑ* = 0.05).

### Possible explanations of the results

The results from this Dream Catcher experiment suggest that the neural correlates of dreaming consciousness, reportedly found in the power spectra of the brain, may not be robust enough to be useful in blind classification, such as attempted here. Due to the challenging nature of our experimental setup, our failure to pass the Dream Catcher test was not in itself a surprising outcome. However, the failure to predict dreamfulness based on the findings of several past studies—particularly at Step 5 where the choice was binary—is an interesting result. We explore several possible explanations for the poor performance.

First, there were several methodological differences between the original studies, which we have referred to, and ours (Table 3). We had fewer participants than Chellappa *et al.* (*N *=* *17), Esposito *et al.* (*N *=* *11), Siclari *et al.* (*N *=* *32), Scarpelli *et al.* (*N *=* *14), and Siclari *et al.* (*N *=* *12), which exposed us to a greater risk of false negative results. Monte Carlo simulations show that our sample size should have allowed us to detect an effect of size of *d *≥* *1.3 with at least 1 − *β* = 0.8 statistical power and *α* = 0.05 false positive rate for one-tailed *t*-tests of *N *=* *9 paired, Gaussian-distributed samples. We recommend that future investigations rely on larger samples in order to properly confirm or disconfirm the findings in our study.

**Table 3.** Comparison of similar studies

Study authors	NREM dream effects	Study details
Siclari *et al.* (2018)	Decreased frequency and amplitude of **slow waves** (0.25–1 s) @ whole brain, esp. **posterior** and **central** areas Increased frequency of fast spindles @ whole brain	Healthy participants, *N* = 14 (12 used) 735 night awakenings (450 used) 252-channel EEG (185 used), unipolar

Siclari *et al.* (2017)	Decreased **low-frequency power** (1–4 Hz) @ **parieto-occipital** areas Increased high-frequency power (20–50 Hz) @ parieto-occipital, lateral frontal, and temporal areas	Experiment 1: Healthy participants, *N* = 32 233 night awakenings (189 used) 256-channel EEG, source-localized

Scarpelli *et al.* (2017)	Decreased **delta power** (0.50–4.75 Hz) @ **left fronto-temporal** areas	Healthy participants, *N* = 14 28 afternoon awakenings 28-channel EEG, unipolar
Chellappa *et al.* (2011)	Decreased **delta power** (1–3 Hz) @ **fronto-central** areas Decreased spindle power (12–15 Hz) @ centro-parietal areas	Healthy participants, *N* = 17 170 all-day nap awakenings (unreported number used) 12-channel EEG, unipolar
Esposito *et al.* (2004)	Decreased alpha power (8–12 Hz) @ frontal, central, and temporo-parietal areas Decreased **delta power** (0.5–4 Hz) @ **fronto-temporal** areas	Healthy participants, *N* = 11 22 night awakenings 19-channel EEG, unipolar

On the technical level, Siclari *et al.*’s (2017) sleep study differed from ours. Whereas they used a 256-channel high-density EEG system, we were limited to a 29-channel system; and instead of dipole current source modelling, we used Perrin’s method of estimating scalp current source density by taking the Laplacian of fitted spherical splines (Perrin *et al.* 1989, 1990 ). Our emulation of Siclari *et al.*’s hot zone measurements was therefore less precise. Our data were similar to Scarpelli *et al.*’s sleep study: they used 28 scalp electrodes with unipolar referencing for their analysis. Esposito *et al.*’s study used fewer yet: 19 electrodes with unipolar referencing.

Another explanation for our results might have to do with extraneous variability in our data. Clustering works by grouping data according to their relative positions in feature space, but if these positions are influenced more by irrelevant factors or random noise than the relevant effect, then clustering would produce noisy or incorrect results. This was readily apparent from the result of Step 1, where clustering in fact grouped cases together by participant identity and not by dreamfulness (Figs 3B and 9). However, even though participant identities were balanced from Step 2 onwards using pairing information, the classification accuracy did not significantly improve. This remained true even after removing independent components from the EEG time series that clustered contrarily to pairing and participant information in Steps 3 and 4. If our analysis failed to find an effect due to the presence of irrelevant factors or noise, it is unlikely due to only inter-participant variability. Assuming that an effect does exist, we would require a larger sample size to measure it.

Lastly, most of the previous studies did not control for complexity of dream reports, but pooled reports varying in complexity into a single group, and even possibly combined dream reports from different times of night without explicit stratified sampling. Such paradigms might confound the neural correlates of dreaming with time-of-night effects (Pivik and Foulkes 1968; Casagrande *et al.* 1996). We defined dreams as any experiences occurring during sleep—an extremely simple and broad definition in comparison to more fine-grained conceptual frameworks of sleep experience (see Windt *et al.* 2016). In our study, all NREM sleep dreams were static (i.e. lacking change or temporal progression), whilst other studies might have treated only complex, dynamic and temporally progressing experiences as genuine instances of dreaming. It is possible that we failed to classify dreamful and dreamless NREM episodes or find similar patterns of results as in previous studies due to differences in how each study defined dreaming and its subtypes. Chellappa *et al.* (2011) asked participants ‘How much did you dream?’ and identified a given report as dreamful when the answer was ‘greatly’, ‘fairly’ or ‘little’, likely including both static and dynamic dream reports in the study sample. Siclari *et al.* (2017) categorized both perceptual and non-perceptual mentation reports as cases of dreaming as long as participants ‘had been experiencing anything’. Scarpelli *et al.* (2017) selected dreams with narrative and temporal properties (Foulkes and Schmidt 1983) and disregarded dreams without recall. On the other hand, dreamless sleep was identified similarly by all these studies as reports of having no experience. Because the Data Team utilized the early night serial-awakening paradigm to selectively target static NREM dreams, this allowed them to reduce the confounds of varying complexity and time-of-night, while retaining the smallest critical difference between no experience and having a minimal degree of experience (i.e. a static dream). Potentially, compared to previous studies, the observable neural difference that could be expected may be much more subtle in our design: that is, the difference between dreamless and dreamful NREM sleep when dreams were limited to static levels of complexity in the early night.

It is theoretically conceivable that we have experiences all the time during sleep, and that reports of dreamless sleep merely reflect the occasional inability to recall these experiences. However, as long as we need to rely on subjective recollections of experiences, there is no reliable empirical test to verify whether reports of non-dreaming reflect a failure to recall previous experience or a truly unconscious state. This is a potential problem that would equally affect all existing studies of the neural correlates of dreaming.

### The relation between dreamfulness and the depth of NREM sleep

Slow waves have been proposed as a possible neural mechanism underlying the suppression of dream experience across sleep stages. During a slow wave event, neuronal populations undergo bistable states, which can impede inter-areal communication of the brain (Pigorini *et al.* 2015). This has then been suggested to lead to the disruption of conscious dream experiences, thus producing dreamlessness (Tononi *et al.* 2016).

Even though a number of studies have reported that NREM sleep dreaming is associated with a decrease of delta power over differing locations in the brain (Esposito *et al.* 2004; Chellappa *et al.* 2011; Siclari *et al.* 2014, 2017, 2018), we were unable to support this finding. While it could be due to an unidentified confound in our experimental design, data or analysis, it is also possible that a relative delta power decrease is not a necessary correlate of dreaming. We propose another possible explanation: that delta power may simply reflect the depth of NREM sleep—itself defined by the relative power of slow waves—rather than dreaming consciousness per se.

NREM Stages 2, 3 and 4 have been classically delineated by the proportion of visually observable delta waves within the course of a 20- or 30-s epoch (Rechtschaffen and Kales 1968). Stage 2 sleep was primarily defined by the presence of sleep spindles and up to 20% prevalence of delta waves; Stage 3 sleep was defined for having 20–50% delta waves; and epochs with >50% delta waves were scored as Stage 4 sleep. Even by these definitions, there is a huge variance in the amount of delta waves not only between sleep stages but also within a given NREM stage. This has become even more amplified by the newer sleep scoring guidelines introduced by the American Academy of Sleep Medicine (Iber *et al.* 2007) with the merging of NREM Stages 3 and 4 into the N3 stage of ‘slow wave sleep’. In this new classification system, Stage N3 no longer reflects the depth of sleep within that particular sleep stage, as delta waves can occur for 20–100% of the duration of an epoch.

Importantly, dream recall is already known to decrease from Stage 2 to Stage 3 and subsequently from former Stage 3 to Stage 4 (Pivik and Foulkes 1968; Pivik 1971; Foulkes 1982; Moffitt 1982; Noreika *et al.* 2009). As these stages were delineated by the amount of delta waves, it is plausible that the link made by the recent studies between dream recall and delta power in fact reflects the older link between dream recall and NREM depth as defined by the progressive substages of NREM sleep. Previous studies, as well as ours, have utilized sleep stage classification as a measure of sleep depth. While most studies controlled for sleep stages in conjunction with dreamful and dreamless reports, they did not control for the variance of SWA within a sleep stage. Thus, the previously reported association between dreaming and EEG delta power may reflect a simple correlation between dream recall and the depth of NREM sleep as defined by the range of SWA that characterizes each sleep stage rather than intrinsic neural mechanisms of dreaming. This might present an explanation for why we were unable to discriminate between dream report conditions when the distribution of NREM sleep depth (as defined by the proportion of delta waves) was minimized.

As an example, Siclari *et al.* (2017) were able to predict dream recall in real time by awakening participants once delta power decreased below an individual threshold (in addition to a gamma increase). We could argue that such awakenings in fact yielded dream reports that took place during relatively shallow NREM sleep, in which case we would already expect to have more reports of dreaming. Such an uneven association between the presence of dream experience and the depth of sleep, even *within* a given NREM sleep stage, is possible when *all* reports of dreaming and dreamless sleep from the same sleep stage are used for EEG analysis. In our study, the confounding factor of sleep stage (or depth) was minimized by using only a small, matched subsample of data (*N *=* *27 + 27) from a larger pool of awakenings (*N *=* *294), and making sure we had equal numbers of Stage 2 and 3 sleep epochs in dreamful and dreamless conditions from the same participant. Further, in our data, the time from the onset of sleep to the first awakening as well as the time spent in a specific sleep stage before awakening were similar between dreamless and dreamful conditions. Assuming that these parameters are highly correlated with the proportion of SWA, the above control measures increased the likelihood that SWA was similar between our dreamful and dreamless epochs [and notably, the proportion of dreamless vs. dreamful reports did not statistically differ in stages N2 or N3 (Noreika *et al.* 2009)]. The reduced possible bias in sleep depth distribution between the conditions neutralized the low-frequency effect and might explain why dreamful vs. dreamless conditions in our sample did not differ regarding low-frequency power.

In addition, we previously found no spectral power difference between earlier and later awakenings during the three to four hour data collection period (Noreika *et al.* 2009). It seemed that either early night sleep within an approximately 3- to 4-h duration of continuous recording time was too short to produce the ‘time of night’ effect of sleep EEG, or that awakenings during early night sleep prevented the normal change from taking place. This suggests that variance in sleep depth as a function of time of the night is unlikely to bias our data. In this regard, the collection of the data only during the first few hours of sleep may be considered an improvement over studies where time-of-night effects (on dream recall or SWA) were not controlled for.

In sum, we proffer that the above mentioned factors might explain why our analyses failed to predict dreamfulness based on low frequency power and that a much larger and significant bias can be expected in studies with less stringent timing of awakenings and longer sessions throughout the night. Our failure also offers the opportunity to speculate on the possibility that delta power decrease may be interpreted as a confound as opposed to true marker of dreaming.

### Pros and cons of the Dream Catcher paradigm

We have found, through the course of our experiment, that the constraints imposed by the Dream Catcher paradigm force the researchers to focus their efforts on a single determination of the data. This mindset is considerably different to the status quo of scientific research nowadays, where one can pursue multiple avenues of investigation, sequentially or simultaneously, and then deal no further with those yielding non-supporting results. Those failed avenues of investigation end up being incompletely explained and typically remain unpublished. By contrast, the Dream Catcher test permits investigating only a single avenue, and the researchers must address its results whether they support the hypothesis or not. It is similar in spirit to the Registered Reports format for science publishing (see Chambers 2013 for an exposition). In addition, blinding the data removes bias by the researchers; unlike in common practice, where the results are always known, researchers that theoretically pass the Dream Catcher test would do so blindly and based on genuine understanding of the neural constituents of consciousness. The Dream Catcher paradigm encourages not only good science but also a critical assessment of the reliability of past findings.

This paradigm is not, however, without costs. Compared to the testing of multiple hypotheses, the data for a Dream Catcher test—in principle—can only be used once per hypothesis, which is relatively inefficient. Even if there were multiple Analysis Teams working in parallel, there would be no way to ensure that they each tested different hypotheses—because any such attempt would undermine the independence of the teams and could reintroduce bias. We addressed this inefficiency problem somewhat in our design by re-evaluating performance iteratively with a gradual removal of blindness. We hoped this would give us insights into the level of information required for successful classification.

Following the completion of this experiment, we once again address the inefficiency problem and propose that multiple hypotheses could be validly tested within this paradigm through the use of unsupervised machine learning algorithms; for example, by setting up separate algorithms to test each hypothesis and disregard information that might otherwise produce experimenter bias. In our study, we started with the assessment of several families of features for clustering consistency, but ultimately submitted our answers based on just one of them. If we had allowed for the submission of multiple answers in parallel for each set of features, we would have been able to broaden the scope of our study. The caveat, however, is that once again the researchers may be tempted to neglect non-supporting results at the next step.

### Future avenues of investigation

The data analysed here constitute only a portion of the total number of awakenings (294) performed and recorded in our sleep study. Now that we have completed the Dream Catcher experiment with all its self-imposed restrictions, we can further investigate the findings of past studies with the data we did not include. This may well reveal that those unsupported effects do in fact exist, but that they did not survive our precursory data selection process. At the time of the submission of this article, we have preregistered an analysis plan for the remaining data of our study, making explicit hypotheses based on the effects that the previous studies have reported.

Additionally, we can check more speculative effects reported in past studies using our expanded data set. Contemporary theories of consciousness suggest that connectivity (or integration) in the brain is necessary for the emergence of consciousness. The past studies reporting low frequency effects did not consider such features; all their features were measures of univariate data, operating on only one channel at a time. To measure connectivity, we must employ features that operate on multivariate data. Phase coherence and cross-correlation, as we have mentioned in the introduction, are well-known examples. More sophisticated measures have been proposed for quantifying consciousness (Barrett and Seth 2011; Schartner *et al.* 2015; Oizumi *et al.*2016a,b; Tegmark 2016; Kim *et al.* 2018), taking inspiration from the integrated information theory of consciousness (Tononi, 2004, 2008; Oizumi *et al.* 2014). These measures are all necessarily multivariate in nature. It is possible that the true correlates of dreaming consciousness in NREM sleep are to be found in multivariate features rather than univariate ones. We intend to investigate this possibility in future work.

## Authors’ contributions

Original idea of the Dream Catcher paradigm: A.R. Original design of the experiment: V.N., A.R., K.V. Experimental data collection: V.N., K.V. Dream content analysis design and sleep stage scoring: V.N., K.V. Data blinding: L.M. Experimental analysis design: W.W., J.W., N.T. PSG data analysis: W.W. First draft prepared by W.W. V.N., A.R., J.W., K.V., N.T. contributed to revision.

## Supplementary Material

niaa006_Supplementary_Data
