# Supplementary material for: The Dream Catcher experiment: blinded analyses failed to detect markers of dreaming consciousness in EEG spectral power
Source: Neurosci Conscious. 2020 Jul 15;2020(1):niaa006. doi: 10.1093/nc/niaa006 (PMC7362719; doi:10.1093/nc/niaa006)
Supplement: niaa006_Supplementary_Data [file niaa006_supplementary_data.zip › DreamCatcher_SupplementaryDocument1_WW_20200310.pdf]

Supplementary Document 1

Sleep Data Collection Methods

Early night serial awakening. The experimental data were collected during four non-consecutive experimental nights, which took place between 10 p.m. and 6 a.m., with at least three nights between any two sessions. The participants were requested to avoid any stimulants, such as caffeine (6 hours prior to the experiment) or over-the-counter medication and alcohol (24 hours prior to the experiment), which was controlled for by asking participants to fill out a questionnaire before each session. The participants slept in a separate room and were observed through a video camera. The team constantly monitored their PSG on a computer screen and communicated with participants through a digital sound system device.

During each experimental night, participants were awakened during the first 3–4 hours on average 8.17 times (*SD* = 1.24, range 6–11), yielding a total of 294 awakenings (9 participants × 4 nights). This early night serial awakening protocol was shown to be an efficient paradigm for collecting large samples of dream reports while maintaining the stability of EEG spectral power measurements throughout the session (Noreika et al., 2009).

The experimenters awakened the participants by playing a beep sound after confirming through online monitoring of PSG that they had been in Stages 2 or 3 of NREM sleep for at least 3 minutes. The guidelines outlined by Rechtschaffen & Kales (1968) were used in sleep stage scoring. Post-hoc examination of the EEG confirmed that most of the awakenings took place during NREM Stages 2–3 (87%) over the 1-minute period before awakening. Given that serial awakenings from NREM sleep acted as selective REM sleep deprivation, episodes of sleep-onset REM occasionally intruded into the normal progression of sleep stages (13%), in which case participants were awakened as usual. Data from REM awakenings were omitted from this study.

Before the experimental session, the participants were instructed in advance that, immediately after being awakened by the sound signal, they were to give a free oral report of "everything that was going through their mind before awakening"; the procedure was practised during the adaptation night. This instruction was not repeated again after the individual awakenings throughout the night, as a non-prompted free dream report was expected to interfere as little as possible with the very delicate process of remembering their experiences. If the experimenter judged that a free report from the participant contained any pre-awakening thoughts or perceptual experiences, the participant was further examined with a pre-recorded set of 21 questions played on a computer via the sound system (see Supplementary Document 2 for detailed instructions given to participants). The questions included inquiries about objects, feelings, self, and the subjective duration of the dream. Several questions aimed to reinforce recall. If the experimenter judged that a free report from the participant contained no pre-awakening thoughts or perceptual experiences, it was followed by three questions regarding the subjective certainty of dreamless sleep. In cases where these three questions prompted the participant to remember any experiences, they were presented with the set of 21 questions concerning the contents of their dream. If necessary, an unstructured interview was conducted at the end to clarify unclear or ambiguous parts of the report. The experimental night was ended at the wish of the participant or when the number of awakenings was satisfactory. All reports, questionnaire answers, and interviews were recorded on a computer.

EEG acquisition. The EEG montage included 21 electrodes placed according to the standard 10-20 system and 4 additional electrodes (TP7, TP8, PO7, PO8) placed according to the 10-10 system (Oostenveld & Praamstra, 2001). All these electrodes were referenced to the right ear mastoid, and the ground electrode was placed on the temple. In addition, two bipolar EOG electrodes were placed near the lateral canthus and the lower eyelid to measure eye movement, and a pair of bipolar electrodes was placed on the mentalis and submentalis muscles to record chin EMG. Recordings were carried out on a SynAmps amplifier and NeuroScan (4.1.1) data acquisition software at a 2,000 Hz sampling rate. EEG data were saved in the frequency band of 0.05–100 Hz (with a 50 Hz notch filter and a gain of 1,000), EOG data in the band of 0.05–30 Hz (gain 1,000), and EMG data in the band of 5–500 Hz (gain 2,500). Electrodes were silver chloride and attached to the skin using Grass EC2 electrode cream.

Content analysis of post-awakening reports. The post-awakening reports and interviews were transcribed for content analysis, consisting of the following two stages.

First, all reports were divided by two independent raters (Master students in psychology) into four categories: 1) dreamless sleep, 2) white dream, 3) uncertain, and 4) dream (following criteria set out in Dement, 1955). Reports were scored as dreamless if the participant was confident they had no experiences right before awakening. Reports were scored as white dreams if the participant strongly felt they had had some experiences right before awakening, but could not recall any specific content. Reports were scored as uncertain if the participant was unsure whether they had been dreaming or had dreamless sleep right before awakening. Finally, reports were scored as dreams if participants reported any experiences (e.g., perceptions in any sensory modality, sensations, thoughts, feelings, and emotions). Inter-rater reliability for the 4-way categorisation of reports was 94% ($\kappa = .92$).

Second, the dream reports were further categorised by the same two independent raters using Orlinsky's Modified Scale for Perceptual Complexity of Dreams (Noreika et al., 2009; Orlinsky, 1962). This scale consists of 7 perceptual complexity categories, ranging from "1=Participant remembers a specific topic but in isolation: a fragmentary percept, unrelated to anything else" to "7=Participant remembers a long, detailed dream in which the whole scene is replaced by other scenery more than once". Categories 1–4 depict static dreams that lack any change or temporal progression, whereas categories 5–7 depict dynamic dreams that contain a change of at least one perceptual experience (Noreika et al., 2009). The inter-rater reliability for the 7-way categorisation of dream reports was 83.8% (weighted $\kappa = .89$), with most errors consisting of reports assigned to adjacent complexity categories. During both stages of content analysis, the two raters discussed scoring disagreements until agreement was achieved, or—in a few cases—a third rater (author VN) was asked to judge which of the two suggested categories was more accurate.

Sleep scoring. Sleep stages were manually scored by authors VN and KV, and each 1-minute EEG recording was scored as three 20-second epochs. Initially, the scorers agreed on 76% of the epochs. For the remaining 24%, the scorers discussed them until they reached a consensus. Scoring criteria defined by Rechtschaffen & Kales (1968) were followed, which allowed for a more fine-grained consideration of the amount of delta waves during slow wave sleep than the most recent sleep scoring guidelines (Berry et al., 2012).
