# Supplementary material for: The Dream Catcher experiment: blinded analyses failed to detect markers of dreaming consciousness in EEG spectral power
Source: Neurosci Conscious. 2020 Jul 15;2020(1):niaa006. doi: 10.1093/nc/niaa006 (PMC7362719; doi:10.1093/nc/niaa006)
Supplement: niaa006_Supplementary_Data [file niaa006_supplementary_data.zip › DreamCatcher_SupplementaryDocument2_WW_20200310.pdf]

Supplementary Document 2

Dream Report Interview Procedure

Preface

The following is an English translation of the original dream report interview procedure instructions in Finnish, used by the Data Team to conduct the post-awakening dream report interviews. Some examples of the dream reports are given in Supplementary Document 3.

Introduction

The aim of this research project is to investigate the neural correlates of dream experiences during NREM sleep. You will spend five nights in the sleep laboratory: one adaptation night during which we will wake you up four times and you can practise dream reporting, and then four experimental nights during which we will wake you up several times (5–10) from early night sleep, both from light sleep and from deep sleep, and request you to report any dream experiences you might have had. Due to the awakenings, your total sleep time will be shorter than usual, and you may feel tired in the morning and the next day. If you wish to terminate the experiment, you can do so at any time.

Wake-up procedure and reporting instructions

When you have fallen asleep and are in a specific sleep stage, we will wake you up with a sound signal. When you hear the sound signal and wake up, try to stay still and calm, as this way your dream recall is least compromised. We will not ask any questions at this point. Your task is to give us a free recall report: tell us whether you remember having a dream experience during sleep and what the experience was like. Tell us every detail you can remember. By dream experience, we mean every image or thought you had in mind just before waking up.

We do not expect you to recall a dream every time we wake you up. For this research project, it is of utmost importance that you honestly report what you remember, and also honestly report if you don’t remember anything.

When you have recited everything you recall of the dream, we will ask further questions. The questions have been pre-recorded, and we will ask all participants the same questions. Therefore, some of the questions may not feel relevant in the context of your dream. Regardless, try to answer as well as you can, and be honest. If you cannot answer a question, let us know that you cannot remember or tell more about the requested detail. You also have the right to protect your privacy: If you do not wish to share a specific detail of your dream, tell us, and we will not ask further questions about this detail.

If you do not recall any experiences, we will also ask some further questions. Again, the questions have been pre-recorded, and we will present the same questions to all participants.

If we wish to know more about your dream than the recorded questions reveal, we may also ask you some specific, unique questions after the recorded questions.

We will record all your answers to be able to later assess them.

The dream interview and how to answer the questions

If you do not recall a dream:

If, after the sound signal, you report that you do not remember any dream experiences, we will ask you the following questions (N1–N3):

<u>N1) How certain, on a scale from 1 to 5, you are that you did not have any dream experiences?</u>

1 = very uncertain
2 = quite uncertain
3 = cannot say
4 = quite certain
5 = very certain

<u>N2) Do you have the impression that you had a dream experience but cannot recall any specific content?</u>

If you have the impression that you were dreaming, but cannot recall any content at all, answer yes. If you are certain that you did not have any dream experiences, answer no.

<u>N3) What is the last thing you recall?</u>

Tell us the last thing you remember. You don't need to know whether this was from before falling asleep or whether it occurred while asleep.

If you recall a dream:

After you have heard the sound signal and you have reported your dream in as much detail as you can remember, we will ask the following questions (P1–P17):

<u>P1) Did you have the experience just before waking up?</u>

Your task is to estimate when the dream took place. Did the sound signal wake you up in the middle of the experience? Answer yes if you feel that this was the case. If you feel that the dream took place earlier and you had no experiences at the time you woke up, answer no.

<u>P2) How certain, on a scale from 1 to 5, are you that the dream experience took place just before you woke up?</u>

1 = very uncertain
2 = quite uncertain
3 = cannot say
4 = quite certain
5 = very certain

<u>P3) Describe the setting where the dream took place.</u>

Your task is to describe the environment in which the dream events occurred. What was the setting like?

P4) List all the objects in your dream.

Your task is to list all the objects you recall appearing in the dream. No object is too irrelevant to be named.

P5) List all the sounds you heard in your dream.

Your task is to list all the sounds you recall hearing in the dream, such as speech, traffic, or music.

P6) List all the characters in your dream, including also those other than human.

Your task is to list all the human and other animate characters you recall appearing in the dream (animals, fantasy figures, etc.).

P7) Describe all the emotions and feelings and moods you experienced in the dream.

Your task is to describe all the emotions, moods, and feelings you recall having in the dream (e.g., happiness, joy, love, affection, sadness, hate, anger, fear, anxiety, disgust, surprise).

If you did not have any emotional experiences in the dream, report that no emotions were present. Then we will move on with the questions. If you recall having experienced at least one emotion in the dream, we will ask you two additional questions (P7a–P7b):

> P7a. What dream event were the emotions related to?
>
> Describe the event, thought, action or character the emotion was related to or in response to. If you had several emotional experiences, list each, and describe the element each emotion was related to.
>
> P7b. Estimate the intensity of the emotion on a scale from 1 to 5.
>
> Your task is to estimate the intensity of the emotion. If you experienced several separate emotions, please state the name of the emotion first and then estimate its intensity.
>
> 1 = very low
> 2 = quite low
> 3 = moderate
> 4 = quite intense
> 5 = very intense

P8) List all the changes that took place in your dream.

If an object changed into another, if something changed in the setting in which the dream took place, or if a new thought or emotional state appeared, list those.

P9) Were you present or embodied in the dream?

Your task is to tell us whether you were present in the dream and participating in the dream events. If you had a body in the dream, answer yes. If you experienced the dream as a

bodiless spectator or an outside observer, like watching a movie, answer no. If you answer yes, we will ask you two additional questions (P9a–P9b):

> P9a. Describe your dream body.
>
> Describe what kind of a body you had in the dream. Was your dream body identical or similar to your physical body? If it was different to your physical body, how was it different? Aim to describe your dream body in as much detail as possible.
>
> P9b. Were you an active participant in the dream or just an observer?
>
> Describe whether you participated actively in the dream events from an embodied perspective (e.g., talking, thinking, moving, looking around) or whether you were merely a passive spectator or observer.

P10) How clear was the perceptual quality in your dream, on a scale from 1 to 5, compared to how you perceive the world when you are awake?

Your task is to estimate how well the perceptual quality of your dream experience matched the perceptual quality of normal waking experiences. Evaluate how clear the visual, auditory, olfactory, gustatory, tactile and kinaesthetic perceptions and sensations were compared to comparable experiences during wakefulness:

The quality of the perceptions and sensations was

1 = very vague and obscure
2 = quite vague and obscure
3 = almost as clear and defined as during wakefulness
4 = equally clear and defined as during wakefulness
5 = extremely clear and defined, more so than during wakefulness

P11) Estimate the duration of your dream experience.

Estimate how many minutes or seconds your dream experience lasted. If you cannot make an estimation, guess.

1 = about few seconds
2 = about half a minute
3 = about one minute
4 = longer than three minutes
5 = longer than ten minutes
6 = longer than half an hour

P12) How certain are you of your answer to the previous question (P11)?

1 = very uncertain
2 = quite uncertain
3 = cannot say
4 = quite certain
5 = very certain

P13) How quick was the passage of time in your dream?

Your task is to evaluate the subjective experience of passage of time in your dream. How quickly did time seem to be passing compared to how time passes during wakefulness? How fast did the objects or characters move or change in your dream compared to wakefulness?

Time passed:

1 = much slower
2 = a bit slower
3 = comparably to waking experiences
4 = a bit faster
5 = a lot faster

P14) How certain are you of your answer to the previous question (P13)?

1 = very uncertain
2 = quite uncertain
3 = cannot say
4 = quite certain
5 = very certain

P15) What were the factors that allowed you to estimate the passage of time in your dream?

P16) Estimate how long you slept.

Estimate how many minutes or hours you had slept (since the last awakening) before we woke you up.

P17) Do you remember anything else?

If you recall anything else that you have not reported yet, please report what these experiences were.

Postface

The following are summary statistics to some answered questions that the reader might find relevant, counted over the 27 dreamful reports used in the Dream Catcher experiment.

P2) How certain, on a scale from 1 to 5, are you that the dream experience took place just before you woke up?

85% very certain or certain
7% cannot say
7% quite uncertain

P10) How clear was the perceptual quality in your dream, on a scale from 1 to 5, compared to how you perceive the world when you are awake?

15% very vague and obscure
30% quite vague and obscure
33% almost as clear and defined as during wakefulness
19% equally clear and defined as during wakefulness
4% extremely clear and defined, more so than during wakefulness

P11) Estimate the duration of your dream experience.

22%	about few seconds
33%	about half a minute
26%	about one minute
11%	longer than three minutes
7%	longer than ten minutes
0%	longer than half an hour
