# Supplementary material for: The Dream Catcher experiment: blinded analyses failed to detect markers of dreaming consciousness in EEG spectral power
Source: Neurosci Conscious. 2020 Jul 15;2020(1):niaa006. doi: 10.1093/nc/niaa006 (PMC7362719; doi:10.1093/nc/niaa006)
Supplement: niaa006_Supplementary_Data [file niaa006_supplementary_data.zip › DreamCatcher_SupplementaryDocument3_WW_20200310.pdf]

Supplementary Document 3

Examples of Dream Reports

The following are examples of subjects' dream reports after being awakened, translated from the original Finnish reports. All reports were scored as static in Orlinsky's scale (score 1–4). Most were composed of several interconnected perceptions (score 3–4), and most also included a unified background scene (score 4).

Subject 4, 3rd experimental night, awakening 8

"There was a dry open field, and a dog on an agility track. On the track, there was a tube and hurdles. I was uncertain what I was supposed to do there."

Orlinsky score = 3

Subject 5, 1st experimental night, awakening 2

"I dreamt about a green car parked on a street. I saw the car from the side; it was bright green, and I heard the engine running."

Orlinsky score = 3

Subject 8, 4th experimental night, awakening 3

"I saw a cafeteria patio on a street."

Orlinsky score = 1

Subject 11, 3rd experimental night, awakening 3

"I was in a harbour, and I think I had loaded something in some boat. It looked like a Greek harbour, with several boats tied to poles. The sun was shining; on the other side was the sea; and on the other, a forest or something. I think my boyfriend and friends were there with me."

Orlinsky score = 4

Subject 15, experimental night 1, awakening 3

"I dreamt I was in this lab, in this room, and about the electrodes in my head. This bed was there, and someone was putting on the electrodes. In the background, there was a sound of someone talking."

Orlinsky score = 4

Subject 16, experimental night 2, awakening 5

"I was at a party—I think it was my graduation party. We were outdoors in a yard; there was grass, and tables with white table cloths; some dishes and a cake on the table. There were a few people there; I heard them talking."

Orlinsky score = 4
