# Supplementary material for: The Dream Catcher experiment: blinded analyses failed to detect markers of dreaming consciousness in EEG spectral power
Source: Neurosci Conscious. 2020 Jul 15;2020(1):niaa006. doi: 10.1093/nc/niaa006 (PMC7362719; doi:10.1093/nc/niaa006)
Supplement: niaa006_Supplementary_Data [file niaa006_supplementary_data.zip › DreamCatcher_SupplementaryDocument4_WW_20200310.pdf]

Supplementary Document 4

Analysis Team Briefing Document

Preface

The following is the briefing document provided by the Data Team to the Analysis Team prior to the commencing the Dream Catcher experiment.

Dream Catcher Experiment: Gradual removal of blindness

17-03-2008, Turku, Finland

Data and general procedure

Our data set comes from 9 subjects with 3 dream and 3 dreamless sleep reports from each: in total, there are 54 EEG recordings.

The data will be provided to you in 5 steps from the most complex to the easiest one; a following step will always take place only when a previous one has been completed and its success rate has been evaluated. With this kind of procedure, we will get several different decisions where the chance level of successful rating can be evaluated. Consequently, we will be able to see whether the EEG contains information about dreaming, and at what level that information is embedded (e.g. at the level of individual pairs of data cases, at the level of single subjects, or at the level of conditions).

Actual EEG data will be provided only for the Step 1 analysis. During Steps 2-5, only new file names will be given, which, in addition to new information, will always retain the previous ID codes of cases, pairs, subjects, etc.

Glossary

Case: a single 1 min pre-awakening EEG recording of either dream or dreamless sleep (provided in Step 1).

Pair: two 1 min pre-awakening EEG recordings of the same subject; one of them is dream and another one dreamless sleep recording (provided in Step 2).

Subject: information about each subject providing 3 pairs of EEG cases (provided in Step 3).

Group: within each subject, two groups of 3 EEG cases are formed; one group consists of 3 dream cases and another group consists of 3 dreamless sleep cases (provided in Step 4).

Condition: the whole data set is divided into two condition with 27 cases in each; one condition has only dreamless sleep cases and another – only dream cases (provided in Step 5).

Step 1: 54 *cases* are provided

DATA:

Data is provided as 54 coded EEG cases: 27 dream and 27 dreamless sleep recordings (see Fig. 1). You remain blind to which EEG case originates from dreaming and which from dreamless sleep. Each subject will provide 6 cases – 3 dreams and 3 dreamless, but information on which cases belong to the same subject won't be given at this stage.

ID01	ID19	ID37
ID02	ID20	ID38
ID03	ID21	ID39
ID04	ID22	ID40
ID05	ID23	ID41
ID06	ID24	ID42
ID07	ID25	ID43
ID08	ID26	ID44
ID09	ID27	ID45
ID10	ID28	ID46
ID11	ID29	ID47
ID12	ID30	ID48
ID13	ID31	ID49
ID14	ID32	ID50
ID15	ID33	ID51
ID16	ID34	ID52
ID17	ID35	ID53
ID18	ID36	ID54

Figure 1. Example of 54 coded files names for the Step 1 analysis.

TASK:

Your task is to decide which EEG case is "dreaming" and which is "dreamless sleep", and to classify the cases better than at the chance level. Decisions must be based on the analysis of individual cases!

RESULTS:

At least 38 cases out of 54 should be correctly identified in order to succeed above chance level. Also, each research group is expected to provide methodological rationale on what basis the decision was made, that is, which features in the EEG predict dreaming or non-dreaming.

Feedback will be given about overall success rate only, not about correct classification of individual cases.

Step 2. Single *pairs* of dream and dreamless sleep are provided

DATA:

Data is provided in 27 coded EEG pairs, with 1 dream and 1 dreamless case in each pair (see Fig. 2). You remain blind to which EEG case originates from dreaming and which from dreamless sleep. Both cases of a single pair come from the same subject. Each subject will provide 3 pairs, but information on which 3 pairs belong to the same subject won't be given.

ID02-P01	ID13-P10	ID15-P19
ID51-P01	ID37-P10	ID22-P19
ID30-P02	ID01-P11	ID23-P20
ID31-P02	ID27-P11	ID36-P20
ID09-P03	ID32-P12	ID20-P21
ID50-P03	ID43-P12	ID38-P21
ID25-P04	ID06-P13	ID11-P22
ID40-P04	ID48-P13	ID45-P22
ID47-P05	ID28-P14	ID04-P23
ID52-P05	ID33-P14	ID54-P23
ID08-P06	ID05-P15	ID03-P24
ID18-P06	ID39-P15	ID16-P24
ID21-P07	ID12-P16	ID10-P25
ID29-P07	ID17-P16	ID41-P25
ID42-P08	ID14-P17	ID19-P26
ID46-P08	ID34-P17	ID49-P26
ID26-P09	ID07-P18	ID44-P27
ID35-P09	ID24-P18	ID53-P27

Figure 2. Example of 54 coded files names for the Step 2 analysis.

TASK:

Your task is to decide which case in each EEG pair is "dreaming" and which is "dreamless sleep", and to classify the cases better than at the chance level. Decisions must be based on the analysis of individual pairs, i.e. independent from other pairs!

RESULTS:

At least 18 pairs out of 27 should be correctly identified in order to succeed above chance level. Also, each research group is expected to provide methodological rationale on what basis the decision was made, that is, which features in the EEG predict dreaming or non-dreaming.

Feedback will be given about overall success rate only, not about correct classification of individual pairs.

Step 3: ***Subject*** **information for pairs is provided**

DATA:

Data is provided in 27 EEG pairs with 1 dream and 1 dreamless case in each, but it is not indicated which is a dreamless and which is a dream case (see Fig. 3). This time, however, 3 pairs of each subject are identified, so that comparison of pairs from the same subject is possible.

ID32-P12-S1	ID09-P03-S4	ID26-P09-S7
ID43-P12-S1	ID50-P03-S4	ID35-P09-S7
ID06-P13-S1	ID47-P05-S4	ID28-P14-S7
ID48-P13-S1	ID52-P05-S4	ID33-P14-S7
ID10-P25-S1	ID07-P18-S4	ID23-P20-S7
ID41-P25-S1	ID24-P18-S4	ID36-P20-S7
ID02-P01-S2	ID30-P02-S5	ID01-P11-S8
ID51-P01-S2	ID31-P02-S5	ID27-P11-S8
ID21-P07-S2	ID42-P08-S5	ID11-P22-S8
ID29-P07-S2	ID46-P08-S5	ID45-P22-S8
ID12-P16-S2	ID05-P15-S5	ID03-P24-S8
ID17-P16-S2	ID39-P15-S5	ID16-P24-S8
ID08-P06-S3	ID13-P10-S6	ID25-P04-S9
ID18-P06-S3	ID37-P10-S6	ID40-P04-S9
ID04-P23-S3	ID14-P17-S6	ID15-P19-S9
ID54-P23-S3	ID34-P17-S6	ID22-P19-S9
ID44-P27-S3	ID19-P26-S6	ID20-P21-S9
ID53-P27-S3	ID49-P26-S6	ID38-P21-S9

Figure 3. Example of 54 coded files names for the Step 3 analysis.

TASK:

Your task is to decide which case in each pair is “dreaming” and which “dreamless sleep”, and to classify the pairs higher than at chance level. Still, decision should be done pairwise only, as no averaging between pairs is possible at this stage (averaging based on correct pooling of the type of cases is made possible at the next stage). At this stage, it is possible to compare EEGs of different individuals, and different pairs within the same individual, which should reduce variability in the data analysis.

RESULTS:

At least 18 pairs out of 27 should be correctly identified in order to succeed above chance level. Also, each research group is expected to provide methodological rationale, on what EEG basis the decision was made, that is, which features in the EEG predict dreaming or non-dreaming.

Feedback will be given about overall success rate only, not about correct classification of individual pairs.

Step 4: *Groups* of cases within each subject are provided

DATA:

Within each subject, two groups of different experimental conditions are provided (see Fig. 4). One group contains 3 EEGs prior to awakenings leading to dream report, and another group has 3 EEG cases from dreamless sleep of the same subject. However, it won't be revealed, which is which. You will get 18 such groups coming from 9 subjects, yet, no identification across all subjects and their groups will be provided.

ID31-P02-S5-G01	ID35-P09-S7-G07	ID30-P02-S5-G13
ID46-P08-S5-G01	ID28-P14-S7-G07	ID42-P08-S5-G13
ID05-P15-S5-G01	ID23-P20-S7-G07	ID39-P15-S5-G13
ID37-P10-S6-G02	ID43-P12-S1-G08	ID50-P03-S4-G14
ID34-P17-S6-G02	ID48-P13-S1-G08	ID47-P05-S4-G14
ID19-P26-S6-G02	ID41-P25-S1-G08	ID07-P18-S4-G14
ID01-P11-S8-G03	ID25-P04-S9-G09	ID02-P01-S2-G15
ID11-P22-S8-G03	ID15-P19-S9-G09	ID29-P07-S2-G15
ID16-P24-S8-G03	ID38-P21-S9-G09	ID12-P16-S2-G15
ID32-P12-S1-G04	ID27-P11-S8-G10	ID08-P06-S3-G16
ID06-P13-S1-G04	ID45-P22-S8-G10	ID54-P23-S3-G16
ID10-P25-S1-G04	ID03-P24-S8-G10	ID53-P27-S3-G16
ID09-P03-S4-G05	ID18-P06-S3-G11	ID40-P04-S9-G17
ID52-P05-S4-G05	ID04-P23-S3-G11	ID22-P19-S9-G17
ID24-P18-S4-G05	ID44-P27-S3-G11	ID20-P21-S9-G17
ID13-P10-S6-G06	ID51-P01-S2-G12	ID26-P09-S7-G18
ID14-P17-S6-G06	ID21-P07-S2-G12	ID33-P14-S7-G18
ID49-P26-S6-G06	ID17-P16-S2-G12	ID36-P20-S7-G18

Figure 4. Example of 54 coded files names for the Step 4 analysis.

TASK:

Your task is to compare within-subject EEG groups, and to decide, for each subject separately, which group is "dreaming", and which is "dreamless sleep". At this stage, 3 vs. 3 EEGs can be grouped together and within-subject averaging can be made. Yet, between-subject comparisons are still impossible.

RESULTS:

At least 14 EEG groups of cases out of 18 should be correctly identified in order to succeed above chance level. Also, each research group is expected to provide methodological rationale, on what EEG basis the decision was made, that is, which features in the EEG predict dreaming or non-dreaming.

Feedback will be given about overall success rate only, not about correct classification of individual groups.

Step 5: *Conditions* of data set are provided

DATA:

Finally, all data will be classified between-subjects into 2 conditions with 27 EEG cases per each (see Fig. 5).

ID01-P11-S8-G03-C1	ID51-P01-S2-G12-C1	ID27-P11-S8-G10-C2
ID11-P22-S8-G03-C1	ID21-P07-S2-G12-C1	ID45-P22-S8-G10-C2
ID16-P24-S8-G03-C1	ID17-P16-S2-G12-C1	ID03-P24-S8-G10-C2
ID32-P12-S1-G04-C1	ID30-P02-S5-G13-C1	ID18-P06-S3-G11-C2
ID06-P13-S1-G04-C1	ID42-P08-S5-G13-C1	ID04-P23-S3-G11-C2
ID10-P25-S1-G04-C1	ID39-P15-S5-G13-C1	ID44-P27-S3-G11-C2
ID09-P03-S4-G05-C1	ID08-P06-S3-G16-C1	ID50-P03-S4-G14-C2
ID52-P05-S4-G05-C1	ID54-P23-S3-G16-C1	ID47-P05-S4-G14-C2
ID24-P18-S4-G05-C1	ID53-P27-S3-G16-C1	ID07-P18-S4-G14-C2
ID13-P10-S6-G06-C1	ID31-P02-S5-G01-C2	ID02-P01-S2-G15-C2
ID14-P17-S6-G06-C1	ID46-P08-S5-G01-C2	ID29-P07-S2-G15-C2
ID49-P26-S6-G06-C1	ID05-P15-S5-G01-C2	ID12-P16-S2-G15-C2
ID35-P09-S7-G07-C1	ID37-P10-S6-G02-C2	ID40-P04-S9-G17-C2
ID28-P14-S7-G07-C1	ID34-P17-S6-G02-C2	ID22-P19-S9-G17-C2
ID23-P20-S7-G07-C1	ID19-P26-S6-G02-C2	ID20-P21-S9-G17-C2
ID25-P04-S9-G09-C1	ID43-P12-S1-G08-C2	ID26-P09-S7-G18-C2
ID15-P19-S9-G09-C1	ID48-P13-S1-G08-C2	ID33-P14-S7-G18-C2
ID38-P21-S9-G09-C1	ID41-P25-S1-G08-C2	ID36-P20-S7-G18-C2

Figure 5. Example of 54 coded files names for the Step 5 analysis.

TASK:

Your task will be to decide, which one of the two conditions is “dreaming” and which is “dreamless sleep”. All possible analysis can be made at this stage, as both within- and between-subjects averaging is possible.

RESULTS:

Each research group will report findings of comparison between these two groups. Unfortunately, correct identification of the groups won’t be accepted as success above chance level anymore at this stage (because there is 50% chance to get it right by guessing).
