# Supplementary material for: The Dream Catcher experiment: blinded analyses failed to detect markers of dreaming consciousness in EEG spectral power
Source: Neurosci Conscious. 2020 Jul 15;2020(1):niaa006. doi: 10.1093/nc/niaa006 (PMC7362719; doi:10.1093/nc/niaa006)
Supplement: niaa006_Supplementary_Data [file niaa006_supplementary_data.zip › DreamCatcher_SupplementaryDocument5_WW_20200310.pdf]

Supplementary Table 5

Table S5.

Balance of NREM Stages between dream report conditions

Participant	Number of NREM Stage 2 epochs	
	Dreamful	Dreamless
1	6	6
2	5	5
3	2	2
4	5	5
5	2	2
6	2	3
7	5	6
8	2	1
9	4	3
Total	33	33

Note. Each participant provided 9 NREM Stage 2–3 epochs for each dream report condition. Therefore, the number of Stage 3 epochs for each participant and condition would be 9 minus the given number of Stage 2 epochs. The total number of all epochs for each condition is 81.
