# Supplementary material for: The Dream Catcher experiment: blinded analyses failed to detect markers of dreaming consciousness in EEG spectral power
Source: Neurosci Conscious. 2020 Jul 15;2020(1):niaa006. doi: 10.1093/nc/niaa006 (PMC7362719; doi:10.1093/nc/niaa006)
Supplement: niaa006_Supplementary_Data [file niaa006_supplementary_data.zip › DreamCatcher_SupplementaryDocument6_WW_20200310.pdf]

Supplementary Document 6

Internal Validation of Combination Clustering

Our modified evidence accumulation clustering method—combination clustering—has been fully described in a separate publication (Wong & Tsuchiya, in review). Prior to performing the blinded analyses of the Dream Catcher experiment, the Analysis Team internally validated their method as reported here. Based on the evidence accumulation clustering method (Fred & Jain, 2005), they demonstrated its utility on a set of electrophysiological recordings different from the Dream Catcher experiment's.

In this proof-of-concept, the Analysis Team designed a test with similar parameters to the Dream Catcher experiment. Author NT prepared and blinded the data—the nature and origin of which author WW was naïve to at the time—and tasked WW to use combination clustering to cluster the data into the two groups underlying the data set's composition. The successful results from this demonstration served to validate its use in the blind classification task of the Dream Catcher experiment.

Method

Data

The experimental setup and collection of data used here have been described in previously published studies: please refer to Baroni et al. (2017) and Haun et al. (2017). The Analysis Team used data consisting of electrocorticogram evoked potentials from epilepsy patient 153, taken during a period of presurgical epilepsy monitoring. From a grid electrode array installed over the right temporal lobe, they used recordings from two bipolar re-referenced channels, found over the ventral fusiform area, that were sensitive to faces. No recordings were performed within 48 hours of a major seizure. Visual stimuli—consisting of upright faces, upside-down faces, houses, line drawings of tools, and Mondrian patterns—were presented in a continuous cycle while the participant fixated on a cross at the centre of the display.

The Analysis Team arranged the data recordings into a set of two groups for four separate decoding experiments—each group was composed of a set of 27 evoked potentials (200 ms or 400 samples at 2034.5 Hz) to either face or non-face stimuli, and from either one or two electrodes, as shown in Table S5.1. The first two data sets, A and B, were expected to be quite challenging. Past work on these data sets showed that an optimal, supervised decoder would be able to classify between the two conditions with accuracies of 80% and 70% (data not shown); hence, perfect clustering would not be expected in this experiment. The latter two data sets, C and D, was expected to be easier because only Mondrian stimuli were used in the non-face conditions.

Table S5.1.

Data sets

Data set	Stimuli	Electrodes	Timing (ms)
A	Face vs. non-face	2 intermixed	100–300
B	Face vs. non-face	2 intermixed	400–600
C	Face vs. Mondrian	Single	100–300

D	Face vs. Mondrian	2 intermixed	400–600

Note. Face stimuli consisted of both upright and inverted faces. Non-face stimuli consisted of houses, tools, and Mondrian images. Timing is relative to visual stimulus onset.

Feature Extraction

Similarly to the first stage of Step 1 of the Dream Catcher experiment, WW performed combination clustering on separate sets of extracted features themed around different methods of analysis. They are summarised in Table S5.2. Some of the methods used by the feature sets were equivalent to those used in the Dream Catcher experiment, and they are detailed in Supplementary Document 7; these include *PowerFine*, (*Ar*)*PermEn*, and (*Ar*)*ApEn*.

Table S5.2.

Feature sets

Feature set name	Theme of analysis	Analysis parameters varied	Number of features
AcRms	Autocorrelation coefficients	Time lag	10
ArApEn	Approximate entropy of autoregression residuals	Autoregression order, embedded dimension, tolerance, time lag	72
ArPermEn	Permutation entropy of autoregression residuals	Autoregression order, permutation order, time lag	48
ArStats	Basic statistics of autoregression residuals	Autoregression order, statistic	15
PowerFineDownsam10	Power spectral density of downsampled data	Frequency bin	10
PowerFineSeg4	Power spectral densities of segmented data	Frequency bin, time segment	100
PowerFine	Power spectral density	Frequency bin	100
PowerLowerSeg4	Power spectral density of segmented data for frequencies up to $^{1}/_{10}{}^{\text{th}}$ of the Nyquist frequency	Frequency bin, time segment	20
RmsSeg4	Root mean square of segmented data	Time segment	4

Clustering Procedure

WW performed combination clustering on the feature sets with the same method parameters as that used in Step 1 of the Dream Catcher experiment, producing two clusters of close-to-equal sizes. The clustering results for each data set were collated into a matrix where each row corresponded to an evoked potential, each column corresponded to a feature set, and the matrix entries were their assigned clusters (cluster assignment values were either 1 or -1).

The final clustering answer for each data set was determined by yet another evidence accumulation clustering where the results of the feature sets constituted the new clustering ensemble. This “evidence accumulation step” proceeded by a principal component analysis

on the results matrix with a quartimax rotation (Kaiser, 1958), taking the eigenvalues of the first principal component, and performing a median split of the evoked potentials based on those values. This produced two clusters each of 27 evoked potentials, which were based on the results of the feature sets with the greatest agreement on cluster membership.

Tasks involving data sets A and B were given at a different time from data sets C and D, and consequently, the actual feature sets used were partially different between them. In the first two, feature sets *AcRms*, *ArApEn*, *ArPermEn*, *ArStats*, *PowerFineDownsam10, PowerFineSeg4*, *PowerFine, PowerLowerSeg4,* and *RmsSeg4* were used. In the last two, *RmsSeg4, PowerFine, PowerFineSeg4, AcRms, ArStats, ArApEn,* and *ArPermEn* were used.

Results and Discussion

Using the consistency metric (Equation 1 of the main article) between the clustered answers and the true conditions, the consistencies for data set A and B were respectively 11% and 4%. An optimal decoder would be expected to produce consistencies of approximately 60% and 40%, respectively. Initial results for data sets C and D were positive. The answers respectively had consistencies of 100% and 41%.

The poor clustering of data sets A and B, due to the heterogeneity of the non-face stimuli, suggested that our method (and perhaps blind clustering in general) is sensitive to the number of underlying clusters in the data. In contrast, data set C was the simplest of the data sets tested, as only Mondrian patterns constituted the non-face condition, and evoked potentials only came from one channel location. Here, perfect clustering was achieved.

Interestingly, the results from data set D—which differed from data set C by the selection of evoked potentials from two channel locations—was suboptimal in the first attempt but, post hoc, found to result in 100% consistency after a small change. Correct clustering was achieved here by choosing to perform the last step of evidence accumulation on the second principal component rather than the first. This suggested that the effectiveness of clustering also depends on the features used.

The Analysis Team interpreted the results as favourable as to the effectiveness of the combination clustering algorithm. The results also provided an indication of its limitations, which was kept in mind during the Dream Catcher experiment.
