# Supplementary material for: The Dream Catcher experiment: blinded analyses failed to detect markers of dreaming consciousness in EEG spectral power
Source: Neurosci Conscious. 2020 Jul 15;2020(1):niaa006. doi: 10.1093/nc/niaa006 (PMC7362719; doi:10.1093/nc/niaa006)
Supplement: niaa006_Supplementary_Data [file niaa006_supplementary_data.zip › DreamCatcher_SupplementaryDocument7_WW_20200310.pdf]

Supplementary Document 7

Themed Feature Set Candidates for Step 1 Blind Classification

The Analysis Team constructed eight sets of features, which were themed around various analyses of time series data. The sets were called: *Power*, *PowerFine*, *ACC*, *PermEn*, *ApEn*, *Siclari*, *EogRms* and *EmgRms*. Their constructions are explained below. As described in the main text, the team eventually chose the *PowerFine* features for Step 1 of blind classification due to its high temporal consistency. See Table S7.1 for a summary.

Table S7.1.

Candidate feature sets of Step 1

Set name	Measure	Channels	Time	Parameters	Total features
Power	PSD	Fp1, Fpz, Fp2, F7, F3, Fz, F4, F8, T3, C3, Cz, C4, T4, TP7, TP8, T5, P3, Pz, P4, T6, PO7, PO8, O1, Oz, O2 (25)	0–60 s (1)	{0.5–4, 4–8, 8–14, 14–40, 40–50} Hz (5)	125
PowerFine	PSD	As above (25)	As above (1)	0–49.5 Hz in 0.5 Hz steps (99)	2,475
ACC	RMS	P3-Fp1, P4-Fp2 (2)	0–60 s in 2-s steps in 2-s windows (30)	(1)	660
	Autocorrelation coefficient	As above (2)	As above (30)	First 10 coefficients (10)	
PermEn	Permutation entropy with autoregressive modelling	As in *Power* (25)	0–60 s (1)	*m* = {2, 3, 4} *t* = {1, 2, 4} *p* = {0, 1, 2} (3^3 = 27)	675
ApEn	Approximate entropy with autoregressive modelling	As in *Power* (25)	As above (1)	*m* = {2, 3} *r* = {0.15, 0.2, 0.25} *t* = {1, 2, 4} *p* = {0, 1, 2} (2 × 3^3 = 54)	1,350
Siclari	PSD average of electrodes	Fp1, Fpz, Fp2, F7, F3, Fz, F4, F8, T3, C3, Cz, C4, T4, TP7, TP8, T5, P3, Pz, P4, T6, PO7, PO8, O1, Oz, O2 (25 → 1)	40–60 s (1)	18–50 Hz (1)	11
	As above	As above (1)	As above (1)	1–12 Hz (1)	
	As above	P3, Pz, O1 (3 → 1)	{40–60, 50–60, 56–60} s (3)	As above (1)	
	As above	Fz, F4, F8, Cz, C4, T4 (6 → 1)	As above (3)	18–50 Hz (1)	
	As above	Cz, C4, T4, Pz, P4 (5 → 1)	58–60 s (1)	25–50 Hz (1)	

	As above	T6<br>(1)	As above<br>(1)	As above<br>(1)	
	As above	T5<br>(1)	As above<br>(1)	As above<br>(1)	
EogRms	RMS	E2-E1<br>(1)	{0–20, 20–40, 40–60} s<br>(3)	(1)	3
EmgRms	RMS	SM-M2<br>(1)	As above<br>(3)	(1)	3

Note. The number of elements in entries of columns *Channel*, *Time*, and *Parameters* are given in parentheses. Their products on each row, summed for each feature set, should result in the number of total features of that feature set.

Power

The Analysis Team constructed the *Power* feature set to provide information about both brain activity levels and locations. The set consisted of PSD estimates for each EEG electrode. They evaluated PSDs over the whole 60-s duration in five frequency bands corresponding to delta (0.5–4 Hz), theta (4–8 Hz), alpha (8–14 Hz), beta (14–40 Hz) and gamma (40–50 Hz) wave activity (Noachtar et al., 1999). The electrodes used were a subset of the 10-20 and 10-10 system electrodes (Chatrian, Lettich, & Nelson, 1985; Klem, Luders, Jasper, & Elger, 1999) totalling 25 in number: Fp1, Fpz, Fp2, F7, F3, Fz, F4, F8, T3, C3, Cz, C4, T4, TP7, TP8, T5, P3, Pz, P4, T6, PO7, PO8, O1, Oz and O2. The feature set in total had 125 features (5 frequency bands × 25 channels) for each case.

The Analysis Team preprocessed the EEG time series data offline using the EEGLAB Matlab toolbox (version 13.5.4b; Delorme & Makeig, 2004). From the raw EEG recordings, they detrended the signals and downsampled them from 2000 Hz sampling rate to 100 Hz. They then applied a finite impulse response high-pass filter, windowed using a Hann window of 31 s, at the cutoff frequency of 0.1 Hz. Finally, they re-referenced the channels to the average common electrode. This preprocessed data is also reused in the methods for other feature sets.

PSDs for each electrode were estimated using a modified Welch’s method (Welch, 1967) with time segments of 6.2 s overlapping by 80%, windowed by Hann windows. The Analysis Team performed fast Fourier transform on each window. The power of a frequency band was taken as the mean power (not log-transformed) across frequency bins, from and including the lower limit of the band, up to and exclusive of the upper limit. The Analysis Team modified Welch’s method by trimming off time segments with values in both the upper and lower 5% range prior to taking the mean power of the remaining time segments for each band.

PowerFine

The *PowerFine* feature set was identical the *Power* feature set, except that the PSDs were calculated for much finer-resolution, more uniformly-spaced frequency bins. This was chosen to offer more complete frequency information. The frequency edges of the bins were from 0 to 49.5 Hz in 0.5 Hz steps. PSDs for each electrode were estimated using the modified Welch’s method with time segments of 9.3 s overlapping by 80%, windowed by Hann windows. The feature set in total had 2,475 features (25 channels × 99 frequencies) extracted for each observation.

ACC

This feature set's name was abbreviated from "autocorrelation coefficients", and consisted of features replicated from a study to assess anaesthetic depth following machine learning through cluster analysis (Thomsen et al., 1991). The performance of their method appeared superior to other single parametric measures of spectral distribution (i.e., median and spectral edge frequencies) suggested for assessing anaesthetic depth at the time. Their features were based on autoregressive modelling of EEG signals in 2-s time windows, taking the form of 10 normalised autocorrelation coefficients and 1 root-mean-square measure (RMS).

To replicate this feature set, the Analysis Team utilised the preprocessed (high passed and re-referenced) EEG data performed for the *Power* feature set. From the preprocessed EEG, two bipolar–re-referenced channels were extracted to summarise the activity of the two hemispheres of the brain: electrodes P3-Fp1 and P4-Fp2. They then further filtered those channels, in following with Thomsen's method, with a pre-emphasising first order high-pass filter at 4.2 Hz, and a fourth order low-pass filter at 25 Hz using Matlab's Butterworth filter design tools. For each channel, the Analysis Team segmented the signal into 30 × 2-s time segments, and calculated the RMS and first 10 normalised sample autocorrelation coefficient lags of each segment. The feature set in total had 660 features (2 bipolar channels × 30 time segments × 11 coefficients) extracted for each observation.

PermEn and ApEn

The Analysis Team explored information theory measures of time series data in the form of entropy: specifically through permutation entropy (Bandt & Pompe, 2002) in the *PermEn* feature set, and approximate entropy (Pincus, Gladstone, & Ehrenkranz, 1991) in the *ApEn* feature set. Entropy in signals is a measure of the complexity of a system from which the signals were taken. In the context of consciousness research, entropy has been proposed as a way to monitor loss of consciousness via anaesthetic depth (Bein, 2006; Liang et al., 2015).

For the *PermEn* feature set, the Analysis Team used a Matlab implementation by Ouyang (2012) to compute measures of permutation entropy. Specifically, for each channel of EEG recording (after re-referencing followed by high-pass filtering, as in *Power* feature set), they extracted permutation entropy for all 27 combinations of three parameters of the analysis: the embedding dimension m (2, 3 or 4), the downsampling time delay t (1, 2 or 4), and autoregressive order p (0, 1 or 2). Parameters m and t were direct inputs for the permutation entropy function. The Analysis Team varied m no higher than 4 so as to conserve computation time. Before computing permutation entropies, they performed autoregressive modelling of order p on single EEG signals, and then subtracted the modelled time course from the EEG signals. This was done to reveal the evolution of a time series that may not be linearly dependent on its immediate past states, which may reflect signals related to consciousness better than the raw time series. The Analysis Team carried out autoregression in Matlab with the function provided by the System Identification Toolbox, using the default forward-backward approach to the least-squares autoregressive fitting algorithm. The feature set in total had 675 features (25 electrodes × 3 embedding dimensions × 3 downsampling delays × 3 autoregressive orders) extracted for each case.

For the *ApEn* feature set, the Analysis Team used a Matlab function implemented by Lee (2012) to calculate approximate entropy. Similarly to *ApEn*, for each preprocessed channel of EEG recording, they extracted approximate entropy for all 54 combinations of four parameters: the embedding dimension m (2 or 3), the filter tolerance r (0.15, 0.2 or 0.25;

relative to each signal's sample standard deviation), the downsampling time delay *t* (1, 2 or 4), and autoregressive order *p* (0, 1 or 2). The values for *m* were typical choices by Pincus et al (1991). Like in *PermEn* the Analysis Team subtracted the pth-order autoregressed time course before computing approximate entropy. The feature set in total had 1,350 features (25 channels × 2 embedding dimensions × 3 filter tolerances × 3 downsampling delays × 3 autoregressive orders) extracted for each observation.

Siclari

The extraction of the *Siclari* feature set was designed to approximate the features described by Siclari, LaRocque, Bernardi, Postle, & Tononi's (2014) preprint of Siclari et al.'s (2017) paper. This distinction is important because some differences exist between those two publications. The findings of the 2017 paper were not used because they were not published at the time of analysis. The preprint reported a number of significant differences in low- and high-frequency activity of specific brain regions, as recorded with high-density EEG (256 channels), correlating with conscious experience during sleep. We based our selection of electrodes on the observations of Figure 1a and Figure 4 from the preprint paper. Due to the technical differences between our protocols, the *Siclari* feature set here does not constitute a direct replication. We note the following differences in our protocols. (a) We had considerably fewer channels recorded (28 vs. 256 electrodes). Owing to this, (b) the Analysis Team deferred from performing source localisation, as its reliability is lowered by decreasing the number of channels, and instead interpolated the activity at the scalp level. For the same reason, (c) they did not perform independent component analysis for the removal of ocular, muscular and cardiac artefacts; cases were already chosen to have a minimum of such artefacts.

The *Siclari* feature set used in Step 1 of the Dream Catcher experiment consisted of these 11 effects: the average power across all electrodes at low- (1–12 Hz) and high-frequencies (18–50 Hz); low-frequency power in "parieto-occipital hotspot" channels over the 20, 10 and 4 s preceding awakening; high-frequency power in "frontal hotspot" channels over the final 20, 10 and 4 s preceding awakening; and high-frequency (25–50 Hz) power over the final 2 s preceding awakening at regions correlated with conscious experience of either "spatial setting", "movement", or "speech". The Analysis Team observed that all these reported regions were not laterally symmetrical and honoured this in their feature extraction procedure.

The Analysis Team utilised the preprocessed EEG data performed for the *Power* feature set and performed further operations in order to derive a scalp current source density estimate (CSD) of the EEG signal. They calculated the CSD using Perrin's method of taking the Laplacian of spherical splines (Perrin et al., 1989; Perrin, Pernier, Bertrand, & Echallier, 1990), as implemented for Matlab in CSD Toolbox (Kayser, 2010; Kayser & Tenke, 2006a, 2006b). The parameters for this method were based on those recommended by Kayser & Tenke (2015): a spline flexibility of 4, and smoothing constant of 10^{-5}. The Analysis Team assumed a head radius of 8.9 cm, and thus produced CSDs for each of the 25 original electrode locations for each case.

PSDs for each electrode were estimated following Siclari et al.'s procedure: using Welch's method with time segments of 2 s overlapping by 50%, windowed by Hamming windows. They performed fast Fourier transform on each window. The power of a frequency band was taken as the mean (not log-transformed) power across frequency bins, inclusive of the band's specified edges.

The 11 features were extracted as follows. Two features of power for low and high frequencies respectively were taken as means across all electrode locations in the bands 1–12 Hz and 18–50 Hz over the final 20 s preceding awakening. Three features of low-frequency, average parieto-occipital power were taken across electrodes P3, Pz and O1 in the band 1–12 Hz over the final 20, 10 and 4 s preceding awakening. Three features of high-frequency, average frontal power were taken across electrodes Fz, F4, F8, Cz, C4 and T4 in the band 18–50 Hz over the final 20, 10 and 4 s preceding awakening. Three features for dream contents were taken: spatial setting content power as the average power over electrodes Cz, C4, T4, Pz and P4 in the band 25–50 Hz for the final 2 s preceding awakening; movement content power as that of electrode T6; and speech content power as that of electrode T5.

EogRms and EmgRms

The *EogRms* and *EmgRms* feature sets contained information about eye movement and muscle tone, measured as RMS, over three time segments.

For *EogRms*, the Analysis Team used electrooculograms, taken as the bipolar re-referenced channel E2-E1, resampled to a rate of 60 Hz from 2000 Hz. They applied a first-order Butterworth high-pass filter with a cutoff frequency of 0.5 Hz, to remove particularly slow voltage changes and not saturate the RMS calculation. Three RMS features were calculated for equally divided time segments: 0–20, 20–40 and 40–60 s from awakening.

For *EmgRms*, the Analysis Team used electromyograms, taken as the bipolar re-referenced channel SM-M2, retaining its 2000 Hz sampling rate and online 5–500 Hz bandpass. Three RMS features were calculated for equally divided time segments as in *EogRms*.
