# Supplementary material for: The Dream Catcher experiment: blinded analyses failed to detect markers of dreaming consciousness in EEG spectral power
Source: Neurosci Conscious. 2020 Jul 15;2020(1):niaa006. doi: 10.1093/nc/niaa006 (PMC7362719; doi:10.1093/nc/niaa006)
Supplement: niaa006_Supplementary_Data [file niaa006_supplementary_data.zip › DreamCatcher_SupplementaryDocument8_WW_20200310.pdf]

Supplementary Document 8

Feature Sets and Sub-Clustering Used in Steps 2–5 of Blind Classification

The Analysis Team tailored and carried out unique procedures for each step of blind classification. A summary of their extracted features for each step is given in Table S8.1, and details are described thereafter.

Table S8.1.

Summary of feature sets and sub-clustering used in the blind classification experiment

Step no.	Features		Total features	Sub-clustering
1	PSD	99 frequency bins (0–49.5 Hz),<br>25 electrodes	2,475	*k*-means
2	PSD	19 logarithmic frequency bins (0–50 Hz),<br>averaged over all 25 electrodes,<br>0–60 s	50	Pairwise orientation
	Siclari	11 features (refer to Supp. Table 1)		
	EOG	2 time segments,<br>5 quantiles,<br>bandpass RMS (1–30 Hz),<br>bipolar electrode E2-E1		
	EMG	2 time segments,<br>5 quantiles,<br>bandpass RMS (5–500 Hz),<br>bipolar electrode SM-M2		
3 (ICA)	PSD	19 logarithmic frequency bins (0–50 Hz),<br>0–60 s	19	*k*-means
3	(Same as Step 2)		50	1) Pooled pairwise orientation<br>2) Participant pairwise orientation<br>3) Pooled *k*-means<br>4) Participant *k*-means
4	(Same as Step 2)		50	1) Pooled pairwise orientation<br>2) Participant pairwise orientation<br>3) Pooled *k*-means<br>4) Participant *k*-means
5	SBP low	1 PSD (1–4 Hz),<br>averaged over 24 CSD locations (C5, C3, C4, C6, CP5, CP3, CP1, CPz, CP2, CP4, CP6, P3, P1, Pz, P2, P4, PO7, PO3, POz, PO4, PO8, O1, Oz, O2),<br>40–60 s	3	None
	SBP high	1 PSD (20–50 Hz),<br>averaged over 23 CSD locations (F3, F1, Fz, F2, F4, FC5, FC3, FC1, FCz, FC2, FC4, FC6, T7, C1, Cz, C2, T8, TP7, TP8, P7, P5, P6, P8),<br>40–60 s		

Scarpelli	1 PSD (0.5–4.75 Hz)<br>averaged over 8 electrodes (C3, CP5, F3, FC1, FC5, Fp1, P7, T7),<br>0–60 s

Note. PSD: power spectral density; ICA: independent component analysis; CSD: current source density.

Step 2

The *PowerFine* feature set used in Step 1 of blind classification consisted of 2,475 features (25 EEG channels × 99 frequencies) that measured only EEG PSDs. The resulting classification accuracy was near chance level. By Step 2, the Analysis team suspected that the poor performance might have resulted partly from the large number of features that they used, causing overfitting of data (Domingos, 2012). They also suspected that their clustering algorithm might have performed better with a more encompassing feature set than one that looked only at EEG. The Analysis Team therefore decided to change the feature set for Step 2 to be more concise and to include information from EMG and EOG (different to that from Step 1).

First, they reduced the number of EEG features from Step 1 by taking the average PSD over all 25 electrodes and reducing the number of frequency bins to 19. For 19 features of average EEG power, the Analysis Team evaluated PSDs for each electrode in 18 frequency bins with edges logarithmically spaced between 1.3^{-3} and 1.3^{15} Hz (approximately 0.46–51 Hz), and a lowermost bin with edges at 0 and 1.3^{-3} Hz. These were evaluated in a way similar to the *Power* feature set in Step 1, with one difference being that the time segments used for Welch's method have lengths of 9.3 s instead of 6.2 s.

Second, to avoid losing all spatially specific EEG information, they added PSD estimates for localised brain areas based on the Siclari et al. (2014) report, identical to the *Siclari* features used in Step 1.

Lastly, they extracted 10 features each for EOG and EMG modalities. The data was preprocessed similarly to *EogRms* and *EmgRms* as in Step 1, but for 2 time segments each instead of 10. Thus, for each of these 30-s segments of EMG or EOG, they computed the RMS values of all consecutive 1-s time windows, and took the 0th, 25th, 50th, 75th, and 100th percentiles of those as features.

In total, the Analysis Team used 50 features for Step 2 (19 from average EEG, 10 from EMG, 10 from EOG, and 11 from Siclari EEG)—an almost 50-fold reduction from Step 1.

Step 3

The analysis in Step 3 followed two stages. In the first stage, to take advantage of the revealed participant grouping, the Analysis Team used independent component analysis (ICA) to remove specific components that were likely to affect incorrect clustering results. They considered a component to cluster incorrectly if any pair of cases were to be clustered together rather than apart. The first stage involved extracting the independent components, testing them for incorrect clustering, removing components that were significantly incorrect, and then recomposing the remaining components back into cases for feature extraction. The second stage consisted of combination clustering using a more elaborate sub-clustering procedure than previous Steps. Both stages will now be described.

ICA

The ICA cleaning stage was performed on each participant-group of cases. See Figure S8.1 for a schematic overview.

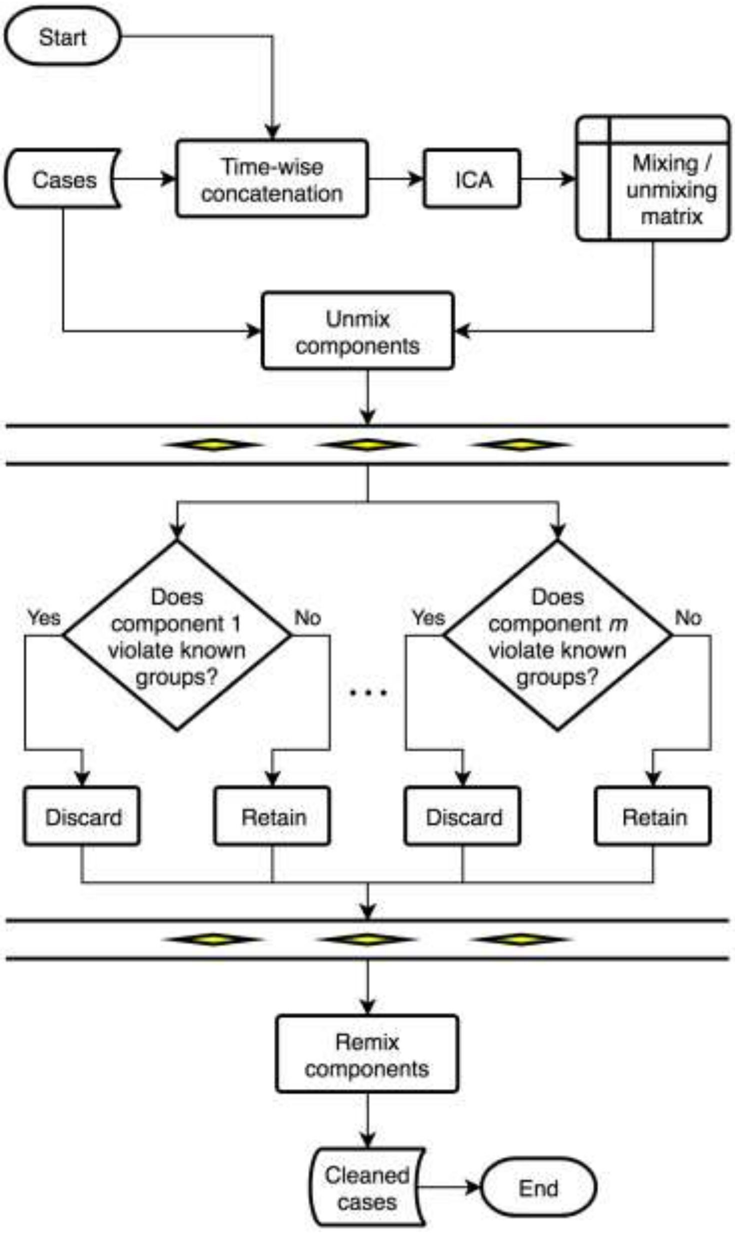

Figure S8.1. Flowchart overview of ICA cleaning. The procedure is performed for each participant-group of cases.

Using the software package *FastICA for Matlab 7.x and 6.x* (version 2.5; Gävert, Hurri, Särelä, & Hyvärinen, 2005), the Analysis Team applied ICA to the combined EEG and EOG time series, concatenating in time across all six cases for a given participant. Independent components were computed using the FastICA "symmetric" approach with a "tanh" nonlinearity function (Rogasch et al., 2014).

At this step of blind classification, case pairing information (that each pair came from the same participant and had different dream report conditions) was already known. Therefore, each of the 26 independent components could be used to cluster the cases of a single participant, and components that reliably clustered contrary to pairing information (i.e., components that represented dissimilar cases as similar) could be identified and removed from the original case recording. The Analysis Team identified these problem components by observing whether they reliably clustered cases of the same pair together rather than apart. To do this, they used a feature set consisting of spectral power in 19 frequency bins, calculated in identical fashion to that in Step 2. Combination clustering was carried out on this feature set extracted for each independent component. Due to the small sample size (three cases) of each participant-group, the Analysis Team tried to avoid overfitting by only sub-clustering in combinations of up to three dimensions, with a *k*-means parameter of $k = 3$, and by not weighting the sub-clustering results by their quality-of-clustering. For this specific case, they also replaced the agglomerative clustering step of combination clustering with one that used an exhaustive cluster configuration search on the co-association similarity matrix, made feasible due to the small sample size per participant-group.

The search objective operationalised by finding the clustering outcome that maximised overall cluster quality as measured by the mean silhouette value (Rousseeuw, 1987). Silhouette values quantify the cohesion of individual members of a cluster and their separation from other clusters, as expressed in the following equation:

$$s(i) = \frac{b(i)-a(i)}{max\{a(i),b(i)\}}, \qquad (1)$$

where $a(i)$ is the average dissimilarity (i.e., distance) of member i to all other members of its cluster, and $b(i)$ is the average dissimilarity of member i to all members of the next nearest cluster.

For the exhaustive search, the Analysis Team restricted the set of possible clusterings to search within by considering only configurations consisting of four clusters: one cluster to represent three cases from one dream report condition group (presumably, the dreamless condition), and the remaining other three clusters to each represent one case from the other dream report condition group. This restriction was imposed to accommodate their conjecture that PSGs accompanying dreamful reports would have a higher variance of their features than those accompanying dreamless reports. There were 20 such configurations (6 choose 3).

An independent component was considered to cluster incorrectly when, after clustering on its extracted feature set, the larger cluster did not consist of exactly one case from each pair. The statistical significance of this effect was measured by bootstrapping the sub-clustering results on which the evidence accumulation clustering was performed. The Analysis Team bootstrapped 1,000 similarity matrices by randomly sampling from the pool of all sub-clustering results with replacement. Formally, they tested the hypothesis that the independent component being considered clustered incorrectly with respect to dream report condition by assuming the null hypothesis that they clustered relevantly to condition. The Analysis Team would reject this null hypothesis if the proportion of bootstrapped clusterings, in which the larger cluster consisted of exactly one case from each pair, was lower than 5%. Significantly incorrect independent components by this criterion were subsequently removed. They then recomposed the EEG and EOG traces from the remaining independent components. On average, 14 components were retained, with a range of 5 to 23.

Sub-clustering. A new set of sub-clustering procedures was devised to take into account the newly revealed participant labels. The Analysis Team could remove unwanted participant effects by sub-clustering cases within each participant; however, this would prevent the analysis from finding similarities amongst cases between participants. The team also suspected that the method of sub-clustering in Step 2—dividing case pairs along the mean orientation of the difference of pairs—may have been discarding other useful information about the cases' actual positions in feature space, as was possible with *k*-means clustering. Therefore, they intended to devise a sub-clustering procedure that additionally retained clustering information from unconstrained case features and from the whole pool of participants.

In fact, the Analysis Team sub-clustered the cases in four different ways and combined the results. In two of the ways, similar to Step 2, they sub-clustered cases in a pairwise manner: firstly with respect to the mean orientation amongst all pairs, and secondly with respect to the mean orientation of each pair's own participant. In the other two ways, they took the method of Step 1's *k*-means sub-clustering and sub-clustered the unpaired cases: firstly amongst all cases, and secondly amongst each participant-group of cases. Therefore, there were four ways which they performed sub-clustering by. The scheme is illustrated in Figure S8.2. After correcting for their average pairwise similarity distance, the Analysis Team averaged those results, thereby performing evidence accumulation, to obtain the final co-association similarity matrix. For per-participant sub-clusterings, they limited the maximum number of features in a combination to 2 due to the effective reduction in data size.

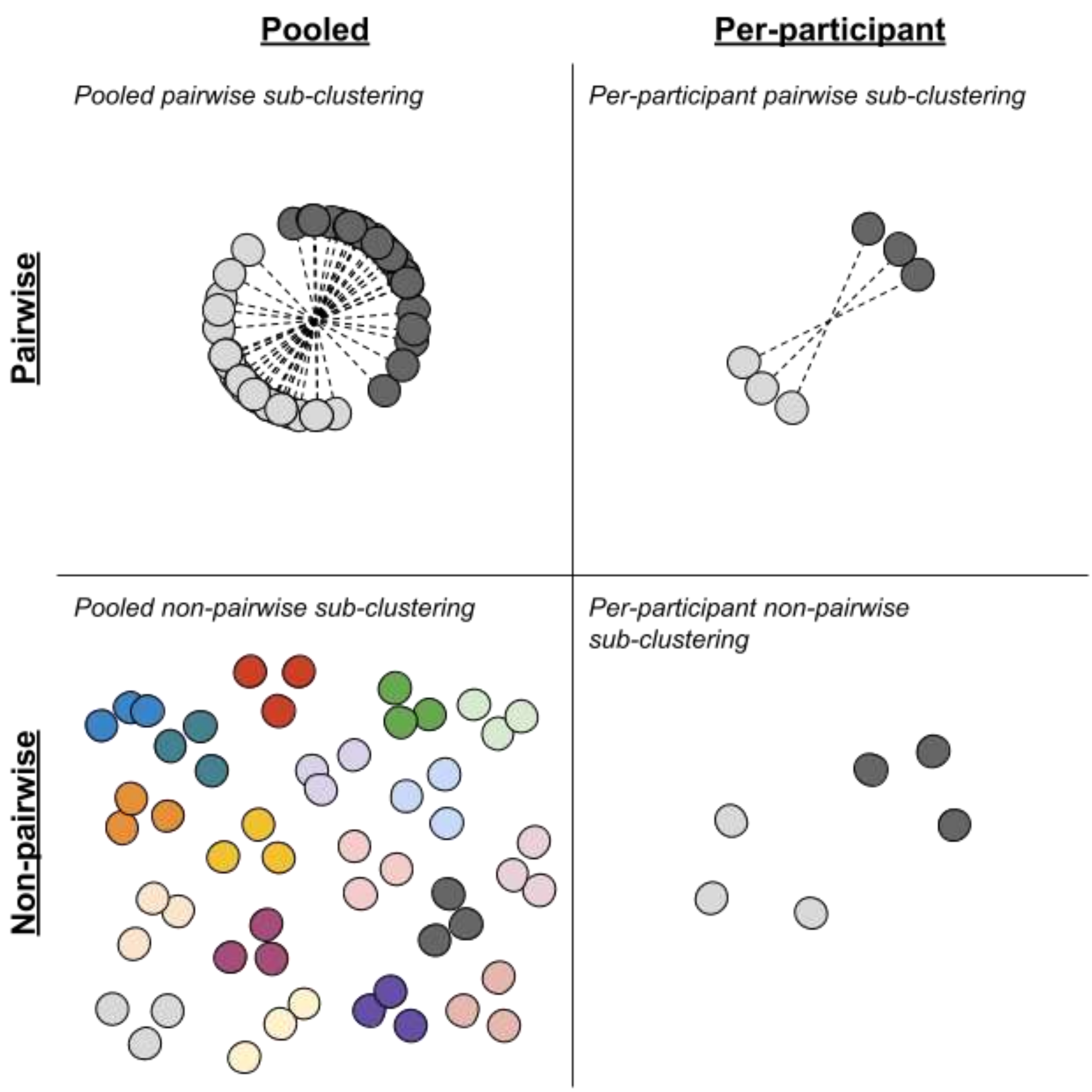

Figure S8.2. Four Step 3 sub-clustering schemes. Each circle corresponds to a case in feature space; each dashed line corresponds to a case pair or difference vector. Colours indicate different sub-clusters that cases belong to. Non-pairwise sub-clustering was implemented as *k*-means clustering.

What follows are the details for each of the four ways of sub-clustering.

Pooled pairwise sub-clustering. For sub-clustering cases in the pooled pairwise manner, the Analysis Team dichotomously grouped each case of every pair with respect to the average pair difference vector orientation across all participants, as done in Step 2. For each sub-clustering's resulting co-association similarity matrix, prior to evidence accumulation, they weighted the matrix such that they took on a mean co-association distance equal to their goodness of clustering value—being 1 minus the mean cosine similarity of the difference vectors. (For more details on the co-association similarity matrix, goodness-of-clustering, and weighting procedures, see Wong & Tsuchiya [in review].)

Pooled non-pairwise sub-clustering. For sub-clustering cases in the pooled non-pairwise manner, the Analysis Team performed k-means clustering over all cases with $k = 18$ (the number of participant-condition groups in our data), after subtracting the means of each participant. For each sub-cluster's resulting co-association similarity matrix, prior to evidence accumulation, they forced any incorrectly co-associated pairs of cases to be recorded as not associated, and then weighted the matrix to take on a mean co-association distance equal to their mean silhouette value.

Per-participant pairwise sub-clustering. For sub-clustering cases in the per-participant pairwise manner, the Analysis Team dichotomously grouped each case of every pair per participant with respect to the participant's average pair difference vector orientation. They weighted each sub-cluster's co-association similarity matrix prior to evidence accumulation just like for pooled pairwise sub-clustering. They then combined the results from all individual participants into the co-association similarity matrix for all cases, where the uncalculated pairwise similarity distances between different participants were simply set to the average of the calculated similarity distances.

Per-participant non-pairwise sub-clustering. For sub-clustering cases in the per-participant non-pairwise manner, the Analysis Team performed k-means clustering on all cases per participant with $k = 4$. They chose $k = 4$ to accommodate their hypothesis that the features of the three dreamful cases might have more variability than the three dreamless cases, and are therefore less clustered. For each sub-cluster's resulting co-association similarity matrix, prior to evidence accumulation, they forced any incorrectly co-associated pairs of cases to be recorded as not associated, then weighted the matrix prior to evidence accumulation just like for per-participant non-pairwise sub-clustering. They then combined the results of all individual participants into the co-association similarity matrix for all cases, just like for per-participant pairwise sub-clustering.

Step 4

Like in Step 3, ICA was performed with the intention to remove specific independent components that were likely to affect incorrect clustering results. Specifically, the Analysis Team tested components for clustering contrary to the revealed participant-condition grouping information in addition to pairing information.

They slightly altered the statistical procedure for testing the significance of bad independent components. Instead of rejecting the null hypothesis if the proportion of valid bootstrapped clusterings was lower than 5%, they calculated a different threshold for each component tested using the Benjamini–Hochberg–Yekutieli procedure (Benjamini & Yekutieli, 2001) to control the false discovery rate to $\alpha = .05$. On average, four components were retained, with a range of one to six.

For the proper combination clustering stage on the recomposed case–averaged participant-condition groups, the Analysis Team sub-clustered these groups as nine pair difference vectors in combinations of up to eight features. They used the same sub-clustering procedure as that of Step 2 on these difference vectors.

Step 5

In Step 5, the Analysis team extracted features based on significant differences reported by Siclari et al. (2017) and Scarpelli et al. (2017). Two features were the low frequency and high

frequency power, which was named respectively *SBP low* and *SBP high* (SBP for the initials of the first three authors of the Siclari et al. paper), and one feature was of the low frequency activity reported by Scarpelli et al., which was named *Scarpelli*.

To replicate the SBP features, the Analysis Team started by downsampling the raw 2000 Hz EEG case recordings to 500 Hz sampling rate and applying a band-pass filter for frequencies 1–50 Hz. The EEG montage was then re-referenced to the common average electrode. They next calculated the CSD using Perrin's method, as described in Supplementary Document 7 for the *Siclari* feature set. Electrodes for *SBP low* were C5, C3, C4, C6, CP5, CP3, CP1, CPz, CP2, CP4, CP6, P3, P1, Pz, P2, P4, PO7, PO3, POz, PO4, PO8, O1, Oz and O2; for *SBP high* were electrodes F3, F1, Fz, F2, F4, FC5, FC3, FC1, FCz, FC2, FC4, FC6, T7, C1, Cz, C2, T8, TP7, TP8, P7, P5, P6 and P8. Electrodes were interpolated where missing using spherical splines. The Analysis Team next estimated the PSDs of each electrode for the 20-s duration until awakening using Welch's method with 2-s Hamming windows and 50% overlap. Finally, for *SBP low*, they took the average power across all its electrodes between 1 and 4 Hz; and for *SBP high*, they took the average power across all its electrodes between 20 and 50 Hz.

To replicate Scarpelli's feature, the Analysis Team started by resampling the raw EEG case recordings down to 250 Hz and applying a band filter for frequencies 0.5–30 Hz. They then retained or interpolated electrodes C3, CP5, F3, FC1, FC5, Fp1, P7 and T7 using the spherical spline method. Note that this region is left-lateralised, consistent with Scarpelli et al.'s reported region of effect. The Analysis Team next estimated PSDs of each electrode, using Welch's method with 4-s rectangular windows and no overlap, for frequencies 0.5 to 4.75 Hz in 0.25 Hz steps. Finally, they calculated the average power across the above-mentioned 8 electrodes and frequencies, and took its natural logarithm as the feature.
